# Supplementary figures and images for: What’s for dinner this time?: DNA authentication of “wild mushrooms” in food products sold in the USA
Source: PeerJ. 2021 Aug 2;9:e11747. doi: 10.7717/peerj.11747 (PMC8340906; doi:10.7717/peerj.11747)

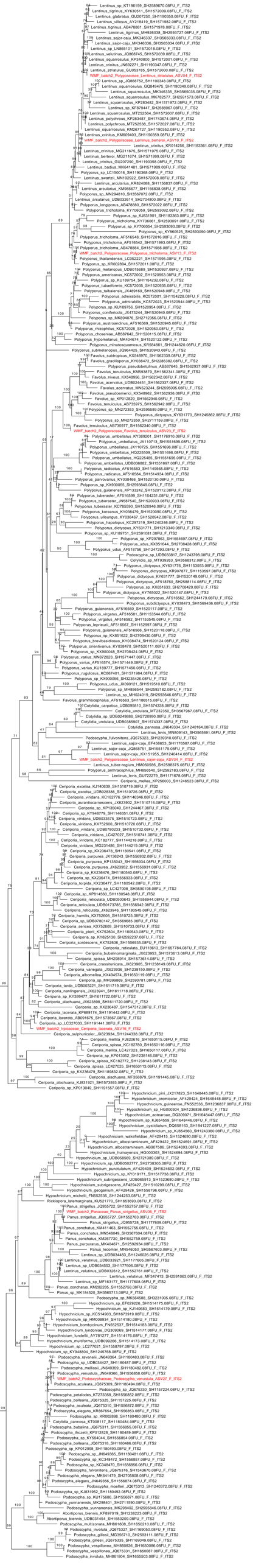

Supplement: Supplemental Information 2 [file peerj-09-11747-s002.zip › sh_general_release_dynamic_s_10.05.2021.POLYPORALES.2.ITS2.mafft.iqtree/210529005243/sh_general_release_dynamic_s_10.05.2021.POLYPORALES.2.ITS2.mafft.fasta.treefile.pdf]

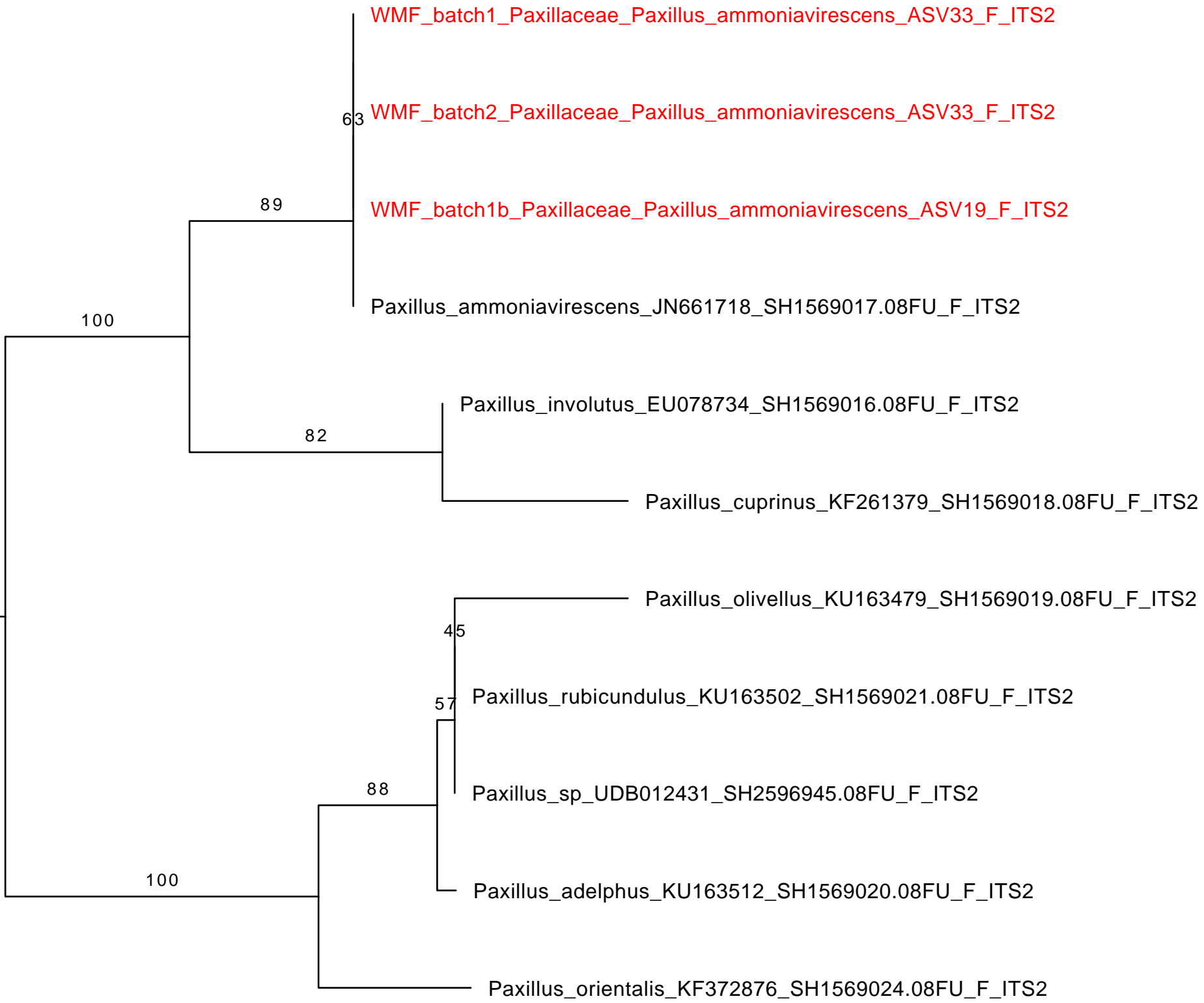

0.02

Supplement: Supplemental Information 2 [file peerj-09-11747-s002.zip › sh_general_release_dynamic_s_10.05.2021.PAXILLUS.2.ITS2.mafft.iqtree/210528002327/sh_general_release_dynamic_s_10.05.2021.PAXILLUS.2.ITS2.mafft.fasta.treefile.pdf]

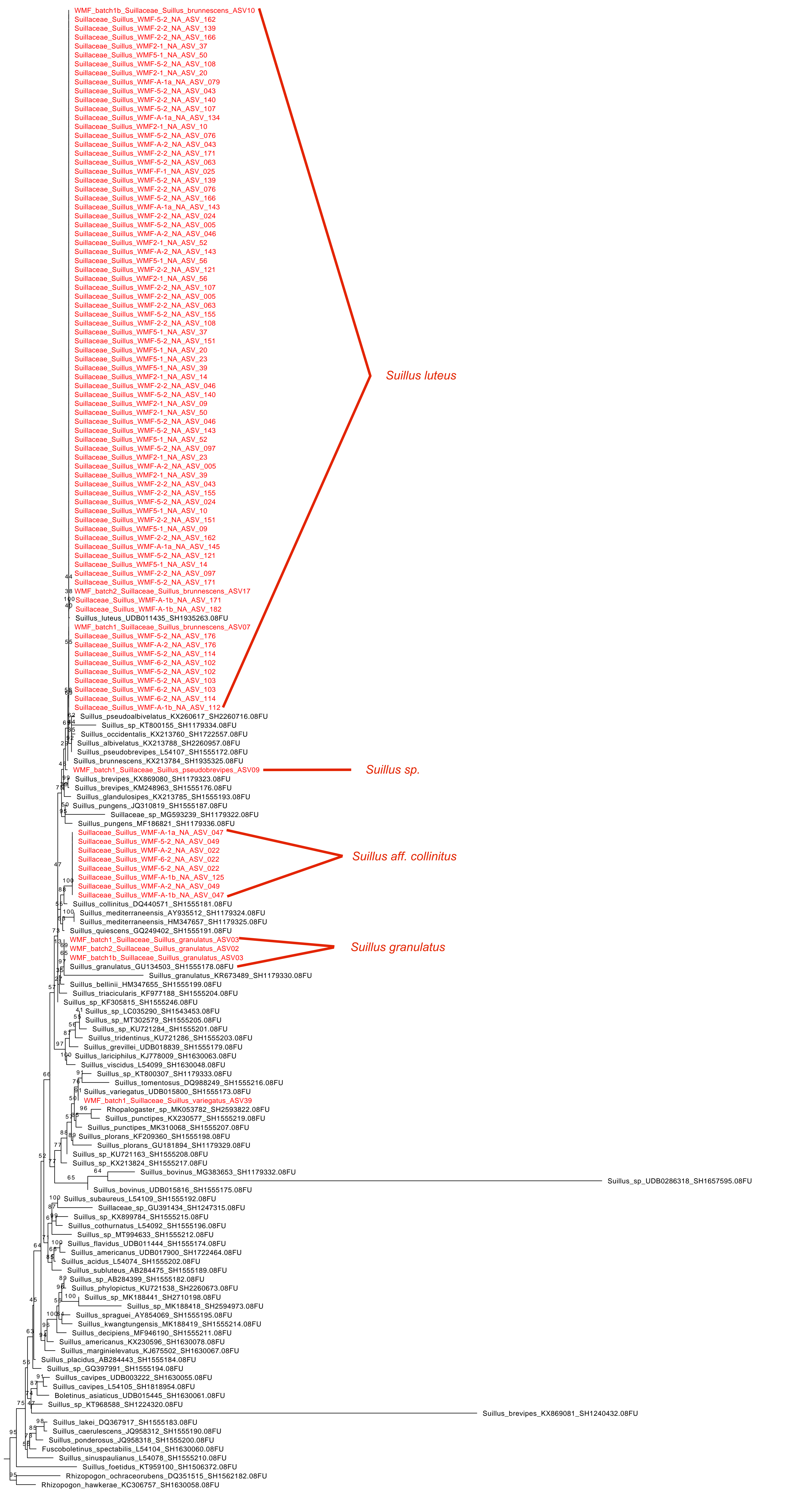

Supplement: Supplemental Information 2 [file peerj-09-11747-s002.zip › sh_general_release_dynamic_s_10.05.2021.SUILLACEAE.3.ITS2.mafft.2.fasta.treefile.pdf]

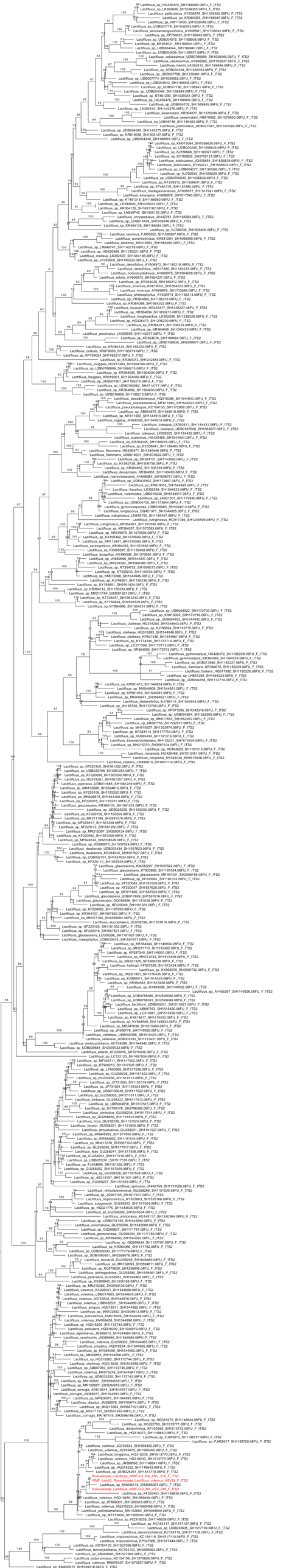

Supplement: Supplemental Information 2 [file peerj-09-11747-s002.zip › sh_general_release_dynamic_s_10.05.2021.LACTIFLUUS.2.ITS2.mafft.fasta.IQTREE/210521213536/sh_general_release_dynamic_s_10.05.2021.LACTIFLUUS.2.ITS2.mafft.fasta.treefile.pdf]

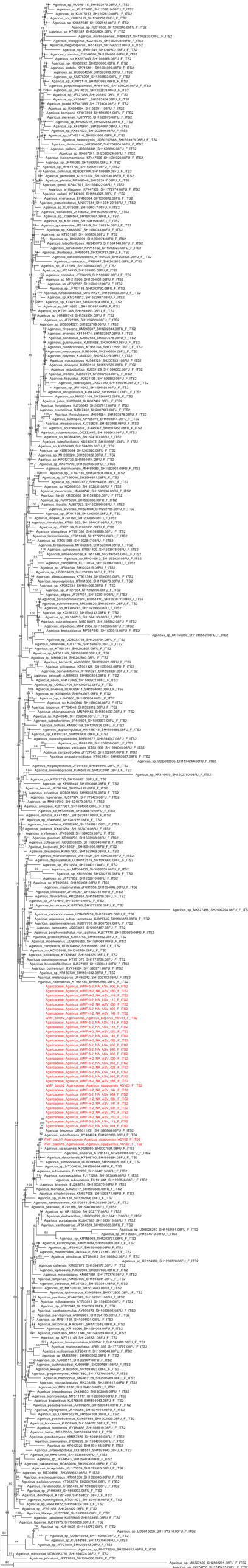

Supplement: Supplemental Information 2 [file peerj-09-11747-s002.zip › sh_general_release_dynamic_s_10.05.2021.AGARICUS.2.ITS2.mafft.IQTREE/210521213349/sh_general_release_dynamic_s_10.05.2021.AGARICUS.2.ITS2.mafft.fasta.treefile.pdf]

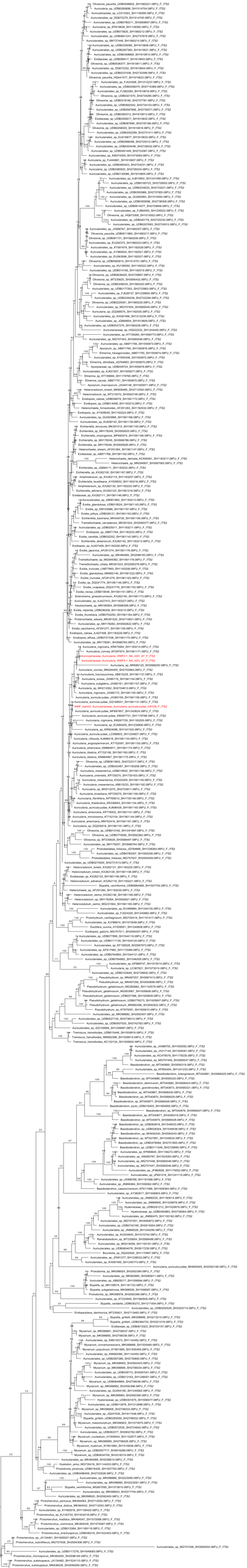

Supplement: Supplemental Information 2 [file peerj-09-11747-s002.zip › AURICULARIA.2.ITS2.mafft.IQTREE/210521213720/sh_general_release_dynamic_s_10.05.2021.AURICULARIA.2.ITS2.mafft.fasta.treefile.pdf]

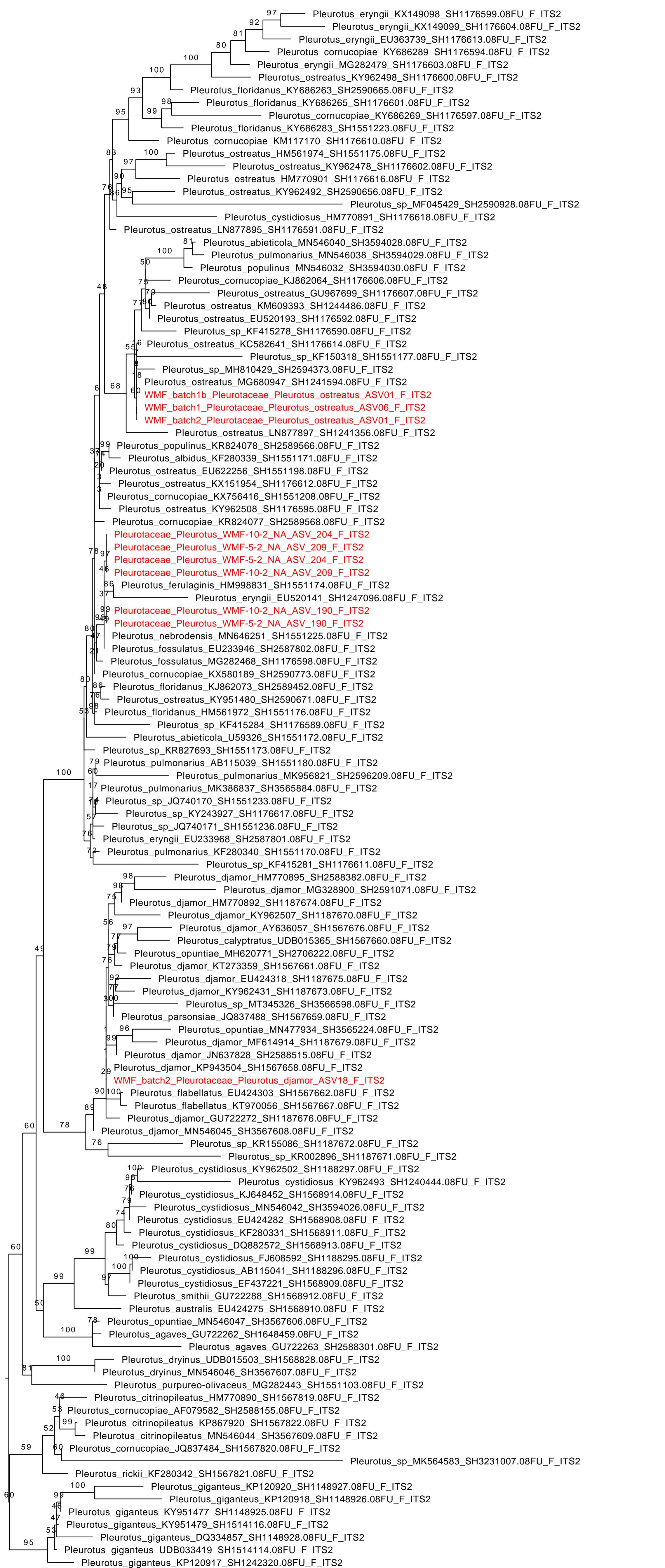

Supplement: Supplemental Information 2 [file peerj-09-11747-s002.zip › sh_general_release_dynamic_s_10.05.2021.PLEUROTUS.2.ITS2.mafft.fasta.treefile.pdf]
