# Supplementary material for: What’s for dinner this time?: DNA authentication of “wild mushrooms” in food products sold in the USA
Source: PeerJ. 2021 Aug 2;9:e11747. doi: 10.7717/peerj.11747 (PMC8340906; doi:10.7717/peerj.11747)
Supplement: Supplemental Information 2 [file sh_general_release_dynamic_s_10.05.2021.RUSSULA.2.ITS2.mafft.fasta.treefile.pdf › sh_general_release_dynamic_s_10.05.2021.RUSSULA.2.ITS2.mafft.fasta.treefile.pdf]

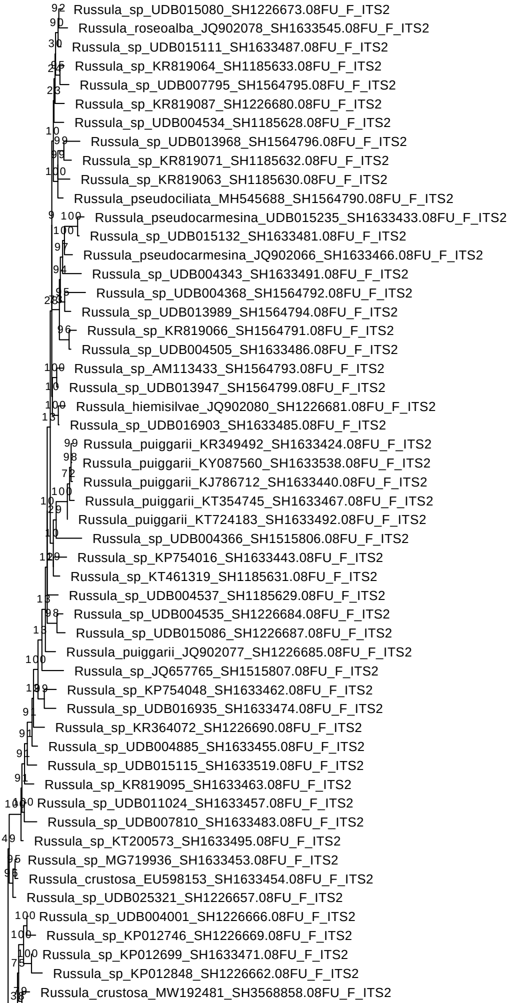
Russula_sp_UDB015080_SH1226673.08FU_F_ITS2
Russula_roseoalba_JQ902078_SH1633545.08FU_F_ITS2
Russula_sp_UDB015111_SH1633487.08FU_F_ITS2
Russula_sp_KR819064_SH1185633.08FU_F_ITS2
Russula_sp_UDB007795_SH1564795.08FU_F_ITS2
Russula_sp_KR819087_SH1226680.08FU_F_ITS2
Russula_sp_UDB004534_SH1185628.08FU_F_ITS2
Russula_sp_UDB013968_SH1564796.08FU_F_ITS2
Russula_sp_KR819071_SH1185632.08FU_F_ITS2
Russula_sp_KR819063_SH1185630.08FU_F_ITS2
Russula_pseudociliata_MH545688_SH1564790.08FU_F_ITS2
Russula_pseudocarmesina_UDB015235_SH1633433.08FU_F_ITS2
Russula_sp_UDB015132_SH1633481.08FU_F_ITS2
Russula_pseudocarmesina_JQ902066_SH1633466.08FU_F_ITS2
Russula_sp_UDB004343_SH1633491.08FU_F_ITS2
Russula_sp_UDB004368_SH1564792.08FU_F_ITS2
Russula_sp_UDB013989_SH1564794.08FU_F_ITS2
Russula_sp_KR819066_SH1564791.08FU_F_ITS2
Russula_sp_UDB004505_SH1633486.08FU_F_ITS2
Russula_sp_AM113433_SH1564793.08FU_F_ITS2
Russula_sp_UDB013947_SH1564799.08FU_F_ITS2
Russula_hiemisilvae_JQ902080_SH1226681.08FU_F_ITS2
Russula_sp_UDB016903_SH1633485.08FU_F_ITS2
Russula_puiggarii_KR349492_SH1633424.08FU_F_ITS2
Russula_puiggarii_KY087560_SH1633538.08FU_F_ITS2
Russula_puiggarii_KJ786712_SH1633440.08FU_F_ITS2
Russula_puiggarii_KT354745_SH1633467.08FU_F_ITS2
Russula_puiggarii_KT724183_SH1633492.08FU_F_ITS2
Russula_sp_UDB004366_SH1515806.08FU_F_ITS2
Russula_sp_KP754016_SH1633443.08FU_F_ITS2
Russula_sp_KT461319_SH1185631.08FU_F_ITS2
Russula_sp_UDB004537_SH1185629.08FU_F_ITS2
Russula_sp_UDB004535_SH1226684.08FU_F_ITS2
Russula_sp_UDB015086_SH1226687.08FU_F_ITS2
Russula_puiggarii_JQ902077_SH1226685.08FU_F_ITS2
Russula_sp_JQ657765_SH1515807.08FU_F_ITS2
Russula_sp_KP754048_SH1633462.08FU_F_ITS2
Russula_sp_UDB016935_SH1633474.08FU_F_ITS2
Russula_sp_KR364072_SH1226690.08FU_F_ITS2
Russula_sp_UDB004885_SH1633455.08FU_F_ITS2
Russula_sp_UDB015115_SH1633519.08FU_F_ITS2
Russula_sp_KR819095_SH1633463.08FU_F_ITS2
Russula_sp_UDB011024_SH1633457.08FU_F_ITS2
Russula_sp_UDB007810_SH1633483.08FU_F_ITS2
Russula_sp_KT200573_SH1633495.08FU_F_ITS2
Russula_sp_MG719936_SH1633453.08FU_F_ITS2
Russula_crustosa_EU598153_SH1633454.08FU_F_ITS2
Russula_sp_UDB025321_SH1226657.08FU_F_ITS2
Russula_sp_UDB004001_SH1226666.08FU_F_ITS2
Russula_sp_KP012746_SH1226669.08FU_F_ITS2
Russula_sp_KP012699_SH1633471.08FU_F_ITS2
Russula_sp_KP012848_SH1226662.08FU_F_ITS2
Russula_crustosa_MW192481_SH3568858.08FU_F_ITS2

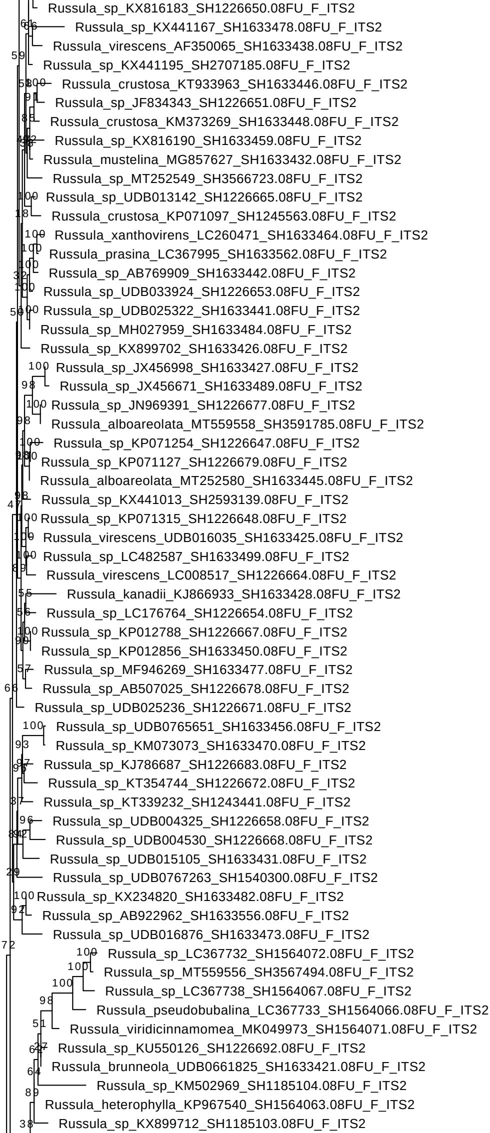
Russula_sp_KX816183_SH1226650.08FU_F_ITS2
Russula_sp_KX441167_SH1633478.08FU_F_ITS2
Russula_virescens_AF350065_SH1633438.08FU_F_ITS2
Russula_sp_KX441195_SH2707185.08FU_F_ITS2
Russula_crustosa_KT933963_SH1633446.08FU_F_ITS2
Russula_sp_JF834343_SH1226651.08FU_F_ITS2
Russula_crustosa_KM373269_SH1633448.08FU_F_ITS2
Russula_sp_KX816190_SH1633459.08FU_F_ITS2
Russula_mustelina_MG857627_SH1633432.08FU_F_ITS2
Russula_sp_MT252549_SH3566723.08FU_F_ITS2
Russula_sp_UDB013142_SH1226665.08FU_F_ITS2
Russula_crustosa_KP071097_SH1245563.08FU_F_ITS2
Russula_xanthovirens_LC260471_SH1633464.08FU_F_ITS2
Russula_prasina_LC367995_SH1633562.08FU_F_ITS2
Russula_sp_AB769909_SH1633442.08FU_F_ITS2
Russula_sp_UDB033924_SH1226653.08FU_F_ITS2
Russula_sp_UDB025322_SH1633441.08FU_F_ITS2
Russula_sp_MH027959_SH1633484.08FU_F_ITS2
Russula_sp_KX899702_SH1633426.08FU_F_ITS2
Russula_sp_JX456998_SH1633427.08FU_F_ITS2
Russula_sp_JX456671_SH1633489.08FU_F_ITS2
Russula_sp_JN969391_SH1226677.08FU_F_ITS2
Russula_alboareolata_MT559558_SH3591785.08FU_F_ITS2
Russula_sp_KP071254_SH1226647.08FU_F_ITS2
Russula_sp_KP071127_SH1226679.08FU_F_ITS2
Russula_alboareolata_MT252580_SH1633445.08FU_F_ITS2
Russula_sp_KX441013_SH2593139.08FU_F_ITS2
Russula_sp_KP071315_SH1226648.08FU_F_ITS2
Russula_virescens_UDB016035_SH1633425.08FU_F_ITS2
Russula_sp_LC482587_SH1633499.08FU_F_ITS2
Russula_virescens_LC008517_SH1226664.08FU_F_ITS2
Russula_kanadii_KJ866933_SH1633428.08FU_F_ITS2
Russula_sp_LC176764_SH1226654.08FU_F_ITS2
Russula_sp_KP012788_SH1226667.08FU_F_ITS2
Russula_sp_KP012856_SH1633450.08FU_F_ITS2
Russula_sp_MF946269_SH1633477.08FU_F_ITS2
Russula_sp_AB507025_SH1226678.08FU_F_ITS2
Russula_sp_UDB025236_SH1226671.08FU_F_ITS2
Russula_sp_UDB0765651_SH1633456.08FU_F_ITS2
Russula_sp_KM073073_SH1633470.08FU_F_ITS2
Russula_sp_KJ786687_SH1226683.08FU_F_ITS2
Russula_sp_KT354744_SH1226672.08FU_F_ITS2
Russula_sp_KT339232_SH1243441.08FU_F_ITS2
Russula_sp_UDB004325_SH1226658.08FU_F_ITS2
Russula_sp_UDB004530_SH1226668.08FU_F_ITS2
Russula_sp_UDB015105_SH1633431.08FU_F_ITS2
Russula_sp_UDB0767263_SH1540300.08FU_F_ITS2
Russula_sp_KX234820_SH1633482.08FU_F_ITS2
Russula_sp_AB922962_SH1633556.08FU_F_ITS2
Russula_sp_UDB016876_SH1633473.08FU_F_ITS2
Russula_sp_LC367732_SH1564072.08FU_F_ITS2
Russula_sp_MT559556_SH3567494.08FU_F_ITS2
Russula_sp_LC367738_SH1564067.08FU_F_ITS2
Russula_pseudobubalina_LC367733_SH1564066.08FU_F_ITS2
Russula_viridicinnamomea_MK049973_SH1564071.08FU_F_ITS2
Russula_sp_KU550126_SH1226692.08FU_F_ITS2
Russula_brunneola_UDB0661825_SH1633421.08FU_F_ITS2
Russula_sp_KM502969_SH1185104.08FU_F_ITS2
Russula_heterophylla_KP967540_SH1564063.08FU_F_ITS2
Russula_sp_KX899712_SH1185103.08FU_F_ITS2

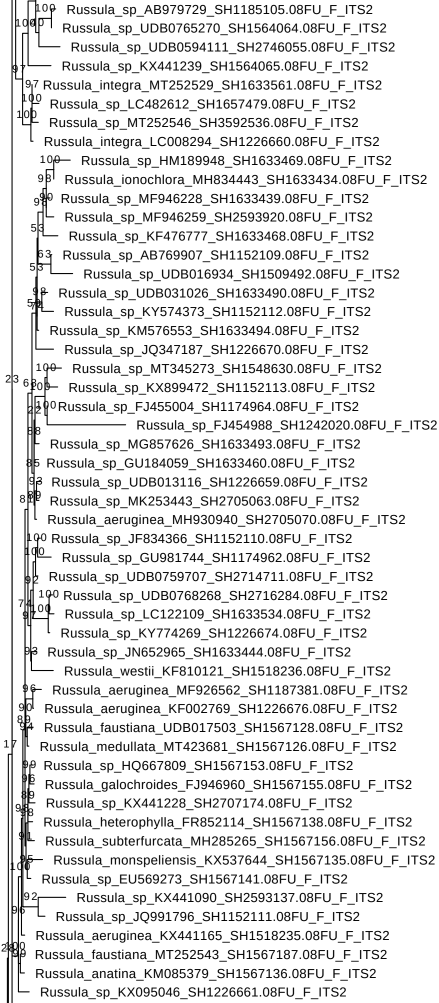
Russula_sp_AB979729_SH1185105.08FU_F_ITS2
Russula_sp_UDB0765270_SH1564064.08FU_F_ITS2
Russula_sp_UDB0594111_SH2746055.08FU_F_ITS2
Russula_sp_KX441239_SH1564065.08FU_F_ITS2
Russula_integra_MT252529_SH1633561.08FU_F_ITS2
Russula_sp_LC482612_SH1657479.08FU_F_ITS2
Russula_sp_MT252546_SH3592536.08FU_F_ITS2
Russula_integra_LC008294_SH1226660.08FU_F_ITS2
Russula_sp_HM189948_SH1633469.08FU_F_ITS2
Russula_ionochlora_MH834443_SH1633434.08FU_F_ITS2
Russula_sp_MF946228_SH1633439.08FU_F_ITS2
Russula_sp_MF946259_SH2593920.08FU_F_ITS2
Russula_sp_KF476777_SH1633468.08FU_F_ITS2
Russula_sp_AB769907_SH1152109.08FU_F_ITS2
Russula_sp_UDB016934_SH1509492.08FU_F_ITS2
Russula_sp_UDB031026_SH1633490.08FU_F_ITS2
Russula_sp_KY574373_SH1152112.08FU_F_ITS2
Russula_sp_KM576553_SH1633494.08FU_F_ITS2
Russula_sp_JQ347187_SH1226670.08FU_F_ITS2
Russula_sp_MT345273_SH1548630.08FU_F_ITS2
Russula_sp_KX899472_SH1152113.08FU_F_ITS2
Russula_sp_FJ455004_SH1174964.08FU_F_ITS2
Russula_sp_FJ454988_SH1242020.08FU_F_ITS2
Russula_sp_MG857626_SH1633493.08FU_F_ITS2
Russula_sp_GU184059_SH1633460.08FU_F_ITS2
Russula_sp_UDB013116_SH1226659.08FU_F_ITS2
Russula_sp_MK253443_SH2705063.08FU_F_ITS2
Russula_aeruginea_MH930940_SH2705070.08FU_F_ITS2
Russula_sp_JF834366_SH1152110.08FU_F_ITS2
Russula_sp_GU981744_SH1174962.08FU_F_ITS2
Russula_sp_UDB0759707_SH2714711.08FU_F_ITS2
Russula_sp_UDB0768268_SH2716284.08FU_F_ITS2
Russula_sp_LC122109_SH1633534.08FU_F_ITS2
Russula_sp_KY774269_SH1226674.08FU_F_ITS2
Russula_sp_JN652965_SH1633444.08FU_F_ITS2
Russula_westii_KF810121_SH1518236.08FU_F_ITS2
Russula_aeruginea_MF926562_SH1187381.08FU_F_ITS2
Russula_aeruginea_KF002769_SH1226676.08FU_F_ITS2
Russula_faustiana_UDB017503_SH1567128.08FU_F_ITS2
Russula_medullata_MT423681_SH1567126.08FU_F_ITS2
Russula_sp_HQ667809_SH1567153.08FU_F_ITS2
Russula_galochroides_FJ946960_SH1567155.08FU_F_ITS2
Russula_sp_KX441228_SH2707174.08FU_F_ITS2
Russula_heterophylla_FR852114_SH1567138.08FU_F_ITS2
Russula_subterfurcata_MH285265_SH1567156.08FU_F_ITS2
Russula_monspeliensis_KX537644_SH1567135.08FU_F_ITS2
Russula_sp_EU569273_SH1567141.08FU_F_ITS2
Russula_sp_KX441090_SH2593137.08FU_F_ITS2
Russula_sp_JQ991796_SH1152111.08FU_F_ITS2
Russula_aeruginea_KX441165_SH1518235.08FU_F_ITS2
Russula_faustiana_MT252543_SH1567187.08FU_F_ITS2
Russula_anatina_KM085379_SH1567136.08FU_F_ITS2
Russula_sp_KX095046_SH1226661.08FU_F_ITS2

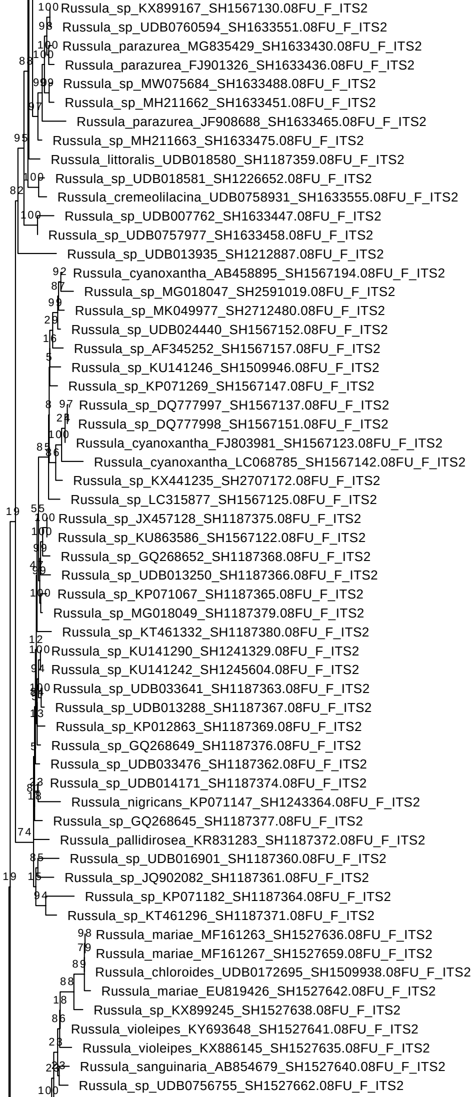
Russula_sp_KX899167_SH1567130.08FU_F_ITS2
Russula_sp_UDB0760594_SH1633551.08FU_F_ITS2
Russula_parazurea_MG835429_SH1633430.08FU_F_ITS2
Russula_parazurea_FJ901326_SH1633436.08FU_F_ITS2
Russula_sp_MW075684_SH1633488.08FU_F_ITS2
Russula_sp_MH211662_SH1633451.08FU_F_ITS2
Russula_parazurea_JF908688_SH1633465.08FU_F_ITS2
Russula_sp_MH211663_SH1633475.08FU_F_ITS2
Russula_littoralis_UDB018580_SH1187359.08FU_F_ITS2
Russula_sp_UDB018581_SH1226652.08FU_F_ITS2
Russula_cremeolilacina_UDB0758931_SH1633555.08FU_F_ITS2
Russula_sp_UDB007762_SH1633447.08FU_F_ITS2
Russula_sp_UDB0757977_SH1633458.08FU_F_ITS2
Russula_sp_UDB013935_SH1212887.08FU_F_ITS2
Russula_cyanoxantha_AB458895_SH1567194.08FU_F_ITS2
Russula_sp_MG018047_SH2591019.08FU_F_ITS2
Russula_sp_MK049977_SH2712480.08FU_F_ITS2
Russula_sp_UDB024440_SH1567152.08FU_F_ITS2
Russula_sp_AF345252_SH1567157.08FU_F_ITS2
Russula_sp_KU141246_SH1509946.08FU_F_ITS2
Russula_sp_KP071269_SH1567147.08FU_F_ITS2
Russula_sp_DQ777997_SH1567137.08FU_F_ITS2
Russula_sp_DQ777998_SH1567151.08FU_F_ITS2
Russula_cyanoxantha_FJ803981_SH1567123.08FU_F_ITS2
Russula_cyanoxantha_LC068785_SH1567142.08FU_F_ITS2
Russula_sp_KX441235_SH2707172.08FU_F_ITS2
Russula_sp_LC315877_SH1567125.08FU_F_ITS2
Russula_sp_JX457128_SH1187375.08FU_F_ITS2
Russula_sp_KU863586_SH1567122.08FU_F_ITS2
Russula_sp_GQ268652_SH1187368.08FU_F_ITS2
Russula_sp_UDB013250_SH1187366.08FU_F_ITS2
Russula_sp_KP071067_SH1187365.08FU_F_ITS2
Russula_sp_MG018049_SH1187379.08FU_F_ITS2
Russula_sp_KT461332_SH1187380.08FU_F_ITS2
Russula_sp_KU141290_SH1241329.08FU_F_ITS2
Russula_sp_KU141242_SH1245604.08FU_F_ITS2
Russula_sp_UDB033641_SH1187363.08FU_F_ITS2
Russula_sp_UDB013288_SH1187367.08FU_F_ITS2
Russula_sp_KP012863_SH1187369.08FU_F_ITS2
Russula_sp_GQ268649_SH1187376.08FU_F_ITS2
Russula_sp_UDB033476_SH1187362.08FU_F_ITS2
Russula_sp_UDB014171_SH1187374.08FU_F_ITS2
Russula_nigricans_KP071147_SH1243364.08FU_F_ITS2
Russula_sp_GQ268645_SH1187377.08FU_F_ITS2
Russula_pallidirosea_KR831283_SH1187372.08FU_F_ITS2
Russula_sp_UDB016901_SH1187360.08FU_F_ITS2
Russula_sp_JQ902082_SH1187361.08FU_F_ITS2
Russula_sp_KP071182_SH1187364.08FU_F_ITS2
Russula_sp_KT461296_SH1187371.08FU_F_ITS2
Russula_mariae_MF161263_SH1527636.08FU_F_ITS2
Russula_mariae_MF161267_SH1527659.08FU_F_ITS2
Russula_chloroides_UDB0172695_SH1509938.08FU_F_ITS2
Russula_mariae_EU819426_SH1527642.08FU_F_ITS2
Russula_sp_KX899245_SH1527638.08FU_F_ITS2
Russula_violeipes_KY693648_SH1527641.08FU_F_ITS2
Russula_violeipes_KX886145_SH1527635.08FU_F_ITS2
Russula_sanguinaria_AB854679_SH1527640.08FU_F_ITS2
Russula_sp_UDB0756755_SH1527662.08FU_F_ITS2

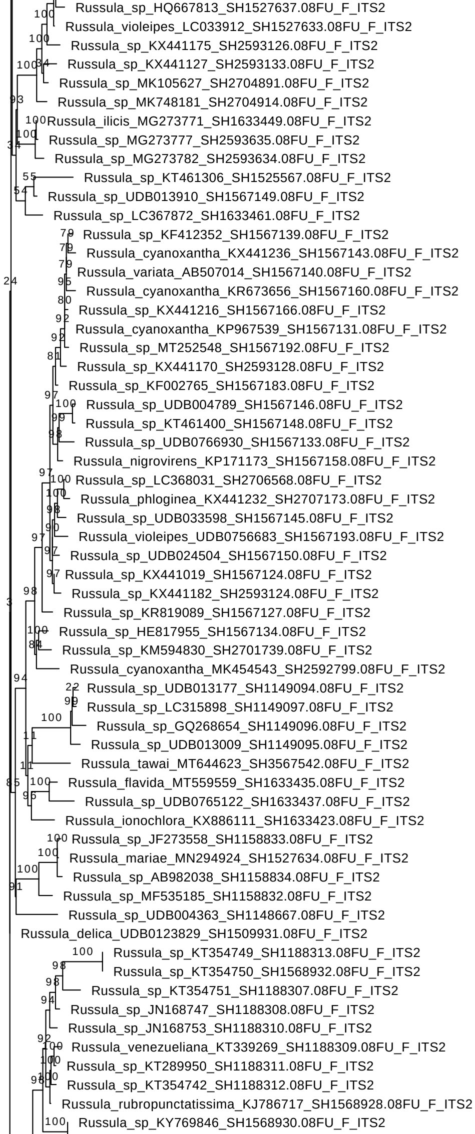
Russula_sp_HQ667813_SH1527637.08FU_F_ITS2
Russula_violeipes_LC033912_SH1527633.08FU_F_ITS2
Russula_sp_KX441175_SH2593126.08FU_F_ITS2
Russula_sp_KX441127_SH2593133.08FU_F_ITS2
Russula_sp_MK105627_SH2704891.08FU_F_ITS2
Russula_sp_MK748181_SH2704914.08FU_F_ITS2
Russula_ilicis_MG273771_SH1633449.08FU_F_ITS2
Russula_sp_MG273777_SH2593635.08FU_F_ITS2
Russula_sp_MG273782_SH2593634.08FU_F_ITS2
Russula_sp_KT461306_SH1525567.08FU_F_ITS2
Russula_sp_UDB013910_SH1567149.08FU_F_ITS2
Russula_sp_LC367872_SH1633461.08FU_F_ITS2
Russula_sp_KF412352_SH1567139.08FU_F_ITS2
Russula_cyanoxantha_KX441236_SH1567143.08FU_F_ITS2
Russula_variata_AB507014_SH1567140.08FU_F_ITS2
Russula_cyanoxantha_KR673656_SH1567160.08FU_F_ITS2
Russula_sp_KX441216_SH1567166.08FU_F_ITS2
Russula_cyanoxantha_KP967539_SH1567131.08FU_F_ITS2
Russula_sp_MT252548_SH1567192.08FU_F_ITS2
Russula_sp_KX441170_SH2593128.08FU_F_ITS2
Russula_sp_KF002765_SH1567183.08FU_F_ITS2
Russula_sp_UDB004789_SH1567146.08FU_F_ITS2
Russula_sp_KT461400_SH1567148.08FU_F_ITS2
Russula_sp_UDB0766930_SH1567133.08FU_F_ITS2
Russula_nigrovirens_KP171173_SH1567158.08FU_F_ITS2
Russula_sp_LC368031_SH2706568.08FU_F_ITS2
Russula_phloginea_KX441232_SH2707173.08FU_F_ITS2
Russula_sp_UDB033598_SH1567145.08FU_F_ITS2
Russula_violeipes_UDB0756683_SH1567193.08FU_F_ITS2
Russula_sp_UDB024504_SH1567150.08FU_F_ITS2
Russula_sp_KX441019_SH1567124.08FU_F_ITS2
Russula_sp_KX441182_SH2593124.08FU_F_ITS2
Russula_sp_KR819089_SH1567127.08FU_F_ITS2
Russula_sp_HE817955_SH1567134.08FU_F_ITS2
Russula_sp_KM594830_SH2701739.08FU_F_ITS2
Russula_cyanoxantha_MK454543_SH2592799.08FU_F_ITS2
Russula_sp_UDB013177_SH1149094.08FU_F_ITS2
Russula_sp_LC315898_SH1149097.08FU_F_ITS2
Russula_sp_GQ268654_SH1149096.08FU_F_ITS2
Russula_sp_UDB013009_SH1149095.08FU_F_ITS2
Russula_tawai_MT644623_SH3567542.08FU_F_ITS2
Russula_flavida_MT559559_SH1633435.08FU_F_ITS2
Russula_sp_UDB0765122_SH1633437.08FU_F_ITS2
Russula_ionochlora_KX886111_SH1633423.08FU_F_ITS2
Russula_sp_JF273558_SH1158833.08FU_F_ITS2
Russula_mariae_MN294924_SH1527634.08FU_F_ITS2
Russula_sp_AB982038_SH1158834.08FU_F_ITS2
Russula_sp_MF535185_SH1158832.08FU_F_ITS2
Russula_sp_UDB004363_SH1148667.08FU_F_ITS2
Russula_delica_UDB0123829_SH1509931.08FU_F_ITS2
Russula_sp_KT354749_SH1188313.08FU_F_ITS2
Russula_sp_KT354750_SH1568932.08FU_F_ITS2
Russula_sp_KT354751_SH1188307.08FU_F_ITS2
Russula_sp_JN168747_SH1188308.08FU_F_ITS2
Russula_sp_JN168753_SH1188310.08FU_F_ITS2
Russula_venezueliana_KT339269_SH1188309.08FU_F_ITS2
Russula_sp_KT289950_SH1188311.08FU_F_ITS2
Russula_sp_KT354742_SH1188312.08FU_F_ITS2
Russula_rubropunctatissima_KJ786717_SH1568928.08FU_F_ITS2
Russula_sp_KY769846_SH1568930.08FU_F_ITS2

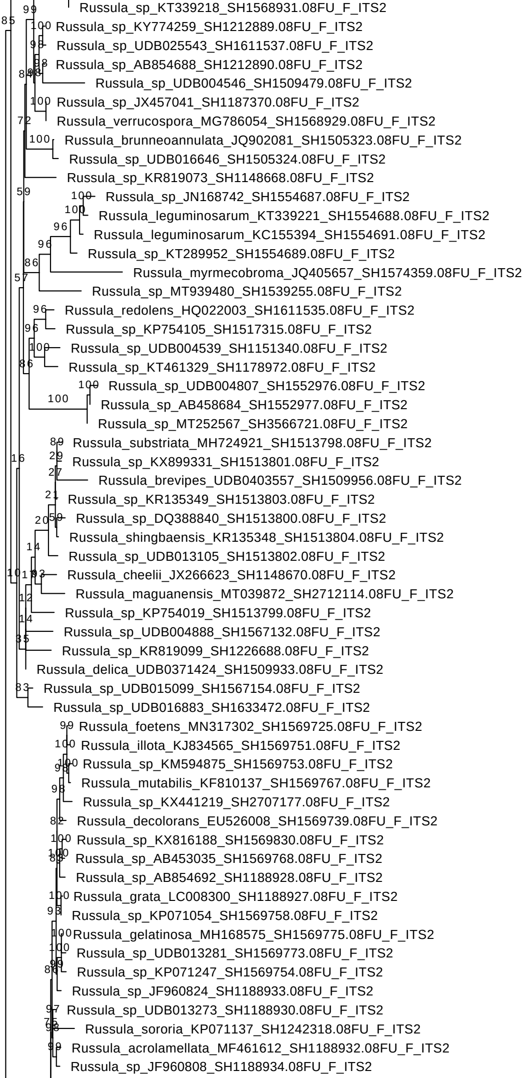
Russula_sp_KT339218_SH1568931.08FU_F_ITS2
Russula_sp_KY774259_SH1212889.08FU_F_ITS2
Russula_sp_UDB025543_SH1611537.08FU_F_ITS2
Russula_sp_AB854688_SH1212890.08FU_F_ITS2
Russula_sp_UDB004546_SH1509479.08FU_F_ITS2
Russula_sp_JX457041_SH1187370.08FU_F_ITS2
Russula_verrucospora_MG786054_SH1568929.08FU_F_ITS2
Russula_brunneoannulata_JQ902081_SH1505323.08FU_F_ITS2
Russula_sp_UDB016646_SH1505324.08FU_F_ITS2
Russula_sp_KR819073_SH1148668.08FU_F_ITS2
Russula_sp_JN168742_SH1554687.08FU_F_ITS2
Russula_leguminosarum_KT339221_SH1554688.08FU_F_ITS2
Russula_leguminosarum_KC155394_SH1554691.08FU_F_ITS2
Russula_sp_KT289952_SH1554689.08FU_F_ITS2
Russula_myrmecobroma_JQ405657_SH1574359.08FU_F_ITS2
Russula_sp_MT939480_SH1539255.08FU_F_ITS2
Russula_redolens_HQ022003_SH1611535.08FU_F_ITS2
Russula_sp_KP754105_SH1517315.08FU_F_ITS2
Russula_sp_UDB004539_SH1151340.08FU_F_ITS2
Russula_sp_KT461329_SH1178972.08FU_F_ITS2
Russula_sp_UDB004807_SH1552976.08FU_F_ITS2
Russula_sp_AB458684_SH1552977.08FU_F_ITS2
Russula_sp_MT252567_SH3566721.08FU_F_ITS2
Russula_substriata_MH724921_SH1513798.08FU_F_ITS2
Russula_sp_KX899331_SH1513801.08FU_F_ITS2
Russula_brevipes_UDB0403557_SH1509956.08FU_F_ITS2
Russula_sp_KR135349_SH1513803.08FU_F_ITS2
Russula_sp_DQ388840_SH1513800.08FU_F_ITS2
Russula_shingbaensis_KR135348_SH1513804.08FU_F_ITS2
Russula_sp_UDB013105_SH1513802.08FU_F_ITS2
Russula_cheelii_JX266623_SH1148670.08FU_F_ITS2
Russula_maguanensis_MT039872_SH2712114.08FU_F_ITS2
Russula_sp_KP754019_SH1513799.08FU_F_ITS2
Russula_sp_UDB004888_SH1567132.08FU_F_ITS2
Russula_sp_KR819099_SH1226688.08FU_F_ITS2
Russula_delica_UDB0371424_SH1509933.08FU_F_ITS2
Russula_sp_UDB015099_SH1567154.08FU_F_ITS2
Russula_sp_UDB016883_SH1633472.08FU_F_ITS2
Russula_foetens_MN317302_SH1569725.08FU_F_ITS2
Russula_illota_KJ834565_SH1569751.08FU_F_ITS2
Russula_sp_KM594875_SH1569753.08FU_F_ITS2
Russula_mutabilis_KF810137_SH1569767.08FU_F_ITS2
Russula_sp_KX441219_SH2707177.08FU_F_ITS2
Russula_decolorans_EU526008_SH1569739.08FU_F_ITS2
Russula_sp_KX816188_SH1569830.08FU_F_ITS2
Russula_sp_AB453035_SH1569768.08FU_F_ITS2
Russula_sp_AB854692_SH1188928.08FU_F_ITS2
Russula_grata_LC008300_SH1188927.08FU_F_ITS2
Russula_sp_KP071054_SH1569758.08FU_F_ITS2
Russula_gelatinosa_MH168575_SH1569775.08FU_F_ITS2
Russula_sp_UDB013281_SH1569773.08FU_F_ITS2
Russula_sp_KP071247_SH1569754.08FU_F_ITS2
Russula_sp_JF960824_SH1188933.08FU_F_ITS2
Russula_sp_UDB013273_SH1188930.08FU_F_ITS2
Russula_sororia_KP071137_SH1242318.08FU_F_ITS2
Russula_acrolamellata_MF461612_SH1188932.08FU_F_ITS2
Russula_sp_JF960808_SH1188934.08FU_F_ITS2

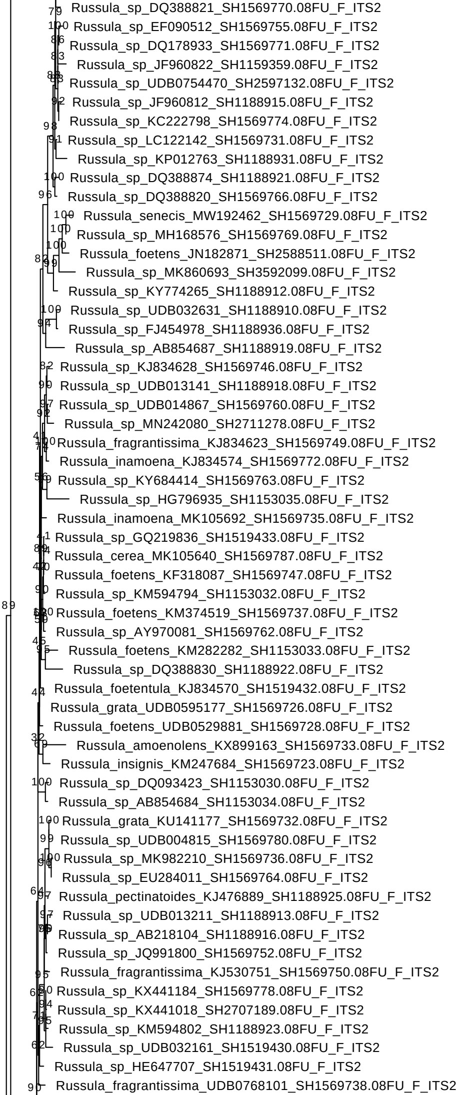
Russula_sp_DQ388821_SH1569770.08FU_F_ITS2
Russula_sp_EF090512_SH1569755.08FU_F_ITS2
Russula_sp_DQ178933_SH1569771.08FU_F_ITS2
Russula_sp_JF960822_SH1159359.08FU_F_ITS2
Russula_sp_UDB0754470_SH2597132.08FU_F_ITS2
Russula_sp_JF960812_SH1188915.08FU_F_ITS2
Russula_sp_KC222798_SH1569774.08FU_F_ITS2
Russula_sp_LC122142_SH1569731.08FU_F_ITS2
Russula_sp_KP012763_SH1188931.08FU_F_ITS2
Russula_sp_DQ388874_SH1188921.08FU_F_ITS2
Russula_sp_DQ388820_SH1569766.08FU_F_ITS2
Russula_senecis_MW192462_SH1569729.08FU_F_ITS2
Russula_sp_MH168576_SH1569769.08FU_F_ITS2
Russula_foetens_JN182871_SH2588511.08FU_F_ITS2
Russula_sp_MK860693_SH3592099.08FU_F_ITS2
Russula_sp_KY774265_SH1188912.08FU_F_ITS2
Russula_sp_UDB032631_SH1188910.08FU_F_ITS2
Russula_sp_FJ454978_SH1188936.08FU_F_ITS2
Russula_sp_AB854687_SH1188919.08FU_F_ITS2
Russula_sp_KJ834628_SH1569746.08FU_F_ITS2
Russula_sp_UDB013141_SH1188918.08FU_F_ITS2
Russula_sp_UDB014867_SH1569760.08FU_F_ITS2
Russula_sp_MN242080_SH2711278.08FU_F_ITS2
Russula_fragrantissima_KJ834623_SH1569749.08FU_F_ITS2
Russula_inamoena_KJ834574_SH1569772.08FU_F_ITS2
Russula_sp_KY684414_SH1569763.08FU_F_ITS2
Russula_sp_HG796935_SH1153035.08FU_F_ITS2
Russula_inamoena_MK105692_SH1569735.08FU_F_ITS2
Russula_sp_GQ219836_SH1519433.08FU_F_ITS2
Russula_cerea_MK105640_SH1569787.08FU_F_ITS2
Russula_foetens_KF318087_SH1569747.08FU_F_ITS2
Russula_sp_KM594794_SH1153032.08FU_F_ITS2
Russula_foetens_KM374519_SH1569737.08FU_F_ITS2
Russula_sp_AY970081_SH1569762.08FU_F_ITS2
Russula_foetens_KM282282_SH1153033.08FU_F_ITS2
Russula_sp_DQ388830_SH1188922.08FU_F_ITS2
Russula_foetentula_KJ834570_SH1519432.08FU_F_ITS2
Russula_grata_UDB0595177_SH1569726.08FU_F_ITS2
Russula_foetens_UDB0529881_SH1569728.08FU_F_ITS2
Russula_amoenolens_KX899163_SH1569733.08FU_F_ITS2
Russula_insignis_KM247684_SH1569723.08FU_F_ITS2
Russula_sp_DQ093423_SH1153030.08FU_F_ITS2
Russula_sp_AB854684_SH1153034.08FU_F_ITS2
Russula_grata_KU141177_SH1569732.08FU_F_ITS2
Russula_sp_UDB004815_SH1569780.08FU_F_ITS2
Russula_sp_MK982210_SH1569736.08FU_F_ITS2
Russula_sp_EU284011_SH1569764.08FU_F_ITS2
Russula_pectinatoides_KJ476889_SH1188925.08FU_F_ITS2
Russula_sp_UDB013211_SH1188913.08FU_F_ITS2
Russula_sp_AB218104_SH1188916.08FU_F_ITS2
Russula_sp_JQ991800_SH1569752.08FU_F_ITS2
Russula_fragrantissima_KJ530751_SH1569750.08FU_F_ITS2
Russula_sp_KX441184_SH1569778.08FU_F_ITS2
Russula_sp_KX441018_SH2707189.08FU_F_ITS2
Russula_sp_KM594802_SH1188923.08FU_F_ITS2
Russula_sp_UDB032161_SH1519430.08FU_F_ITS2
Russula_sp_HE647707_SH1519431.08FU_F_ITS2
Russula_fragrantissima_UDB0768101_SH1569738.08FU_F_ITS2

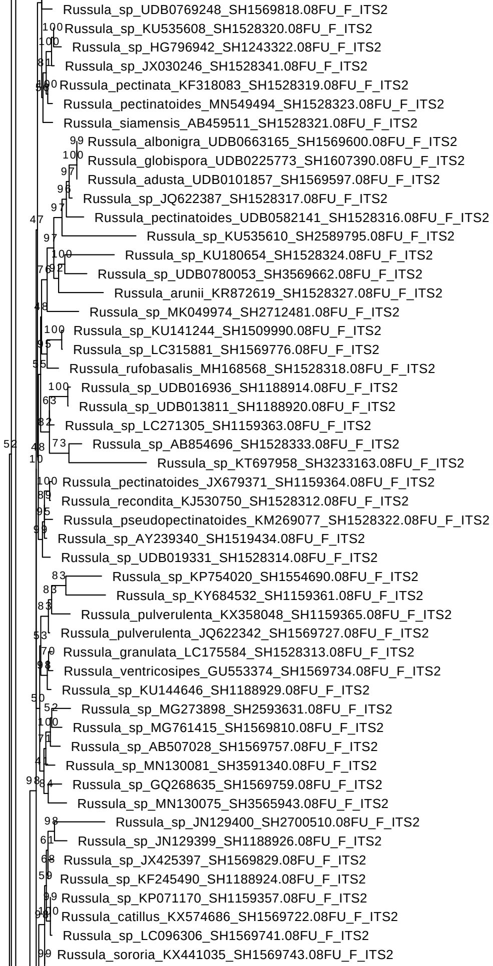
Russula_sp_UDB0769248_SH1569818.08FU_F_ITS2
Russula_sp_KU535608_SH1528320.08FU_F_ITS2
Russula_sp_HG796942_SH1243322.08FU_F_ITS2
Russula_sp_JX030246_SH1528341.08FU_F_ITS2
Russula_pectinata_KF318083_SH1528319.08FU_F_ITS2
Russula_pectinatoides_MN549494_SH1528323.08FU_F_ITS2
Russula_siamensis_AB459511_SH1528321.08FU_F_ITS2
Russula_albonigra_UDB0663165_SH1569600.08FU_F_ITS2
Russula_globispora_UDB0225773_SH1607390.08FU_F_ITS2
Russula_adusta_UDB0101857_SH1569597.08FU_F_ITS2
Russula_sp_JQ622387_SH1528317.08FU_F_ITS2
Russula_pectinatoides_UDB0582141_SH1528316.08FU_F_ITS2
Russula_sp_KU535610_SH2589795.08FU_F_ITS2
Russula_sp_KU180654_SH1528324.08FU_F_ITS2
Russula_sp_UDB0780053_SH3569662.08FU_F_ITS2
Russula_arunii_KR872619_SH1528327.08FU_F_ITS2
Russula_sp_MK049974_SH2712481.08FU_F_ITS2
Russula_sp_KU141244_SH1509990.08FU_F_ITS2
Russula_sp_LC315881_SH1569776.08FU_F_ITS2
Russula_rufobasalis_MH168568_SH1528318.08FU_F_ITS2
Russula_sp_UDB016936_SH1188914.08FU_F_ITS2
Russula_sp_UDB013811_SH1188920.08FU_F_ITS2
Russula_sp_LC271305_SH1159363.08FU_F_ITS2
Russula_sp_AB854696_SH1528333.08FU_F_ITS2
Russula_sp_KT697958_SH3233163.08FU_F_ITS2
Russula_pectinatoides_JX679371_SH1159364.08FU_F_ITS2
Russula_recondita_KJ530750_SH1528312.08FU_F_ITS2
Russula_pseudopectinatoides_KM269077_SH1528322.08FU_F_ITS2
Russula_sp_AY239340_SH1519434.08FU_F_ITS2
Russula_sp_UDB019331_SH1528314.08FU_F_ITS2
Russula_sp_KP754020_SH1554690.08FU_F_ITS2
Russula_sp_KY684532_SH1159361.08FU_F_ITS2
Russula_pulverulenta_KX358048_SH1159365.08FU_F_ITS2
Russula_pulverulenta_JQ622342_SH1569727.08FU_F_ITS2
Russula_granulata_LC175584_SH1528313.08FU_F_ITS2
Russula_ventricosipes_GU553374_SH1569734.08FU_F_ITS2
Russula_sp_KU144646_SH1188929.08FU_F_ITS2
Russula_sp_MG273898_SH2593631.08FU_F_ITS2
Russula_sp_MG761415_SH1569810.08FU_F_ITS2
Russula_sp_AB507028_SH1569757.08FU_F_ITS2
Russula_sp_MN130081_SH3591340.08FU_F_ITS2
Russula_sp_GQ268635_SH1569759.08FU_F_ITS2
Russula_sp_MN130075_SH3565943.08FU_F_ITS2
Russula_sp_JN129400_SH2700510.08FU_F_ITS2
Russula_sp_JN129399_SH1188926.08FU_F_ITS2
Russula_sp_JX425397_SH1569829.08FU_F_ITS2
Russula_sp_KF245490_SH1188924.08FU_F_ITS2
Russula_sp_KP071170_SH1159357.08FU_F_ITS2
Russula_catillus_KX574686_SH1569722.08FU_F_ITS2
Russula_sp_LC096306_SH1569741.08FU_F_ITS2
Russula_sororia_KX441035_SH1569743.08FU_F_ITS2

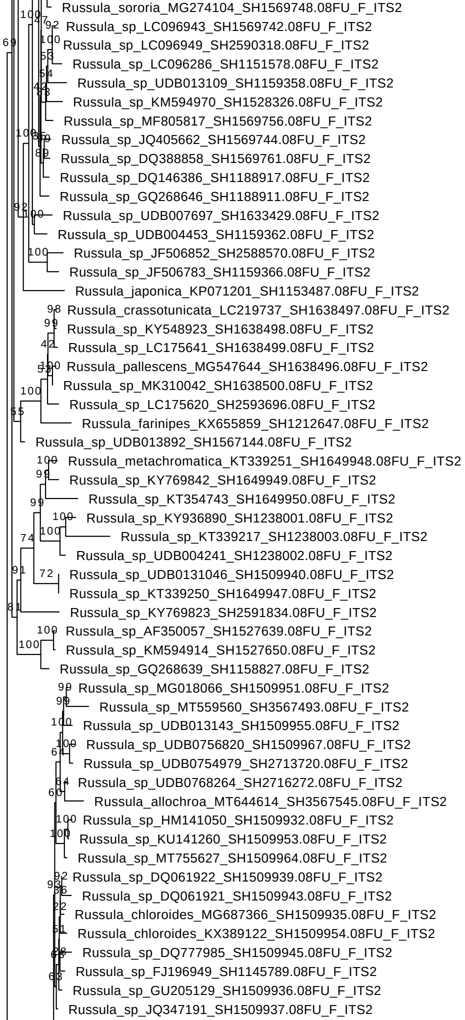
Russula_sororia_MG274104_SH1569748.08FU_F_ITS2
Russula_sp_LC096943_SH1569742.08FU_F_ITS2
Russula_sp_LC096949_SH2590318.08FU_F_ITS2
Russula_sp_LC096286_SH1151578.08FU_F_ITS2
Russula_sp_UDB013109_SH1159358.08FU_F_ITS2
Russula_sp_KM594970_SH1528326.08FU_F_ITS2
Russula_sp_MF805817_SH1569756.08FU_F_ITS2
Russula_sp_JQ405662_SH1569744.08FU_F_ITS2
Russula_sp_DQ388858_SH1569761.08FU_F_ITS2
Russula_sp_DQ146386_SH1188917.08FU_F_ITS2
Russula_sp_GQ268646_SH1188911.08FU_F_ITS2
Russula_sp_UDB007697_SH1633429.08FU_F_ITS2
Russula_sp_UDB004453_SH1159362.08FU_F_ITS2
Russula_sp_JF506852_SH2588570.08FU_F_ITS2
Russula_sp_JF506783_SH1159366.08FU_F_ITS2
Russula_japonica_KP071201_SH1153487.08FU_F_ITS2
Russula_crassotunicata_LC219737_SH1638497.08FU_F_ITS2
Russula_sp_KY548923_SH1638498.08FU_F_ITS2
Russula_sp_LC175641_SH1638499.08FU_F_ITS2
Russula_pallescens_MG547644_SH1638496.08FU_F_ITS2
Russula_sp_MK310042_SH1638500.08FU_F_ITS2
Russula_sp_LC175620_SH2593696.08FU_F_ITS2
Russula_farinipes_KX655859_SH1212647.08FU_F_ITS2
Russula_sp_UDB013892_SH1567144.08FU_F_ITS2
Russula_metachromatica_KT339251_SH1649948.08FU_F_ITS2
Russula_sp_KY769842_SH1649949.08FU_F_ITS2
Russula_sp_KT354743_SH1649950.08FU_F_ITS2
Russula_sp_KY936890_SH1238001.08FU_F_ITS2
Russula_sp_KT339217_SH1238003.08FU_F_ITS2
Russula_sp_UDB004241_SH1238002.08FU_F_ITS2
Russula_sp_UDB0131046_SH1509940.08FU_F_ITS2
Russula_sp_KT339250_SH1649947.08FU_F_ITS2
Russula_sp_KY769823_SH2591834.08FU_F_ITS2
Russula_sp_AF350057_SH1527639.08FU_F_ITS2
Russula_sp_KM594914_SH1527650.08FU_F_ITS2
Russula_sp_GQ268639_SH1158827.08FU_F_ITS2
Russula_sp_MG018066_SH1509951.08FU_F_ITS2
Russula_sp_MT559560_SH3567493.08FU_F_ITS2
Russula_sp_UDB013143_SH1509955.08FU_F_ITS2
Russula_sp_UDB0756820_SH1509967.08FU_F_ITS2
Russula_sp_UDB0754979_SH2713720.08FU_F_ITS2
Russula_sp_UDB0768264_SH2716272.08FU_F_ITS2
Russula_allochroa_MT644614_SH3567545.08FU_F_ITS2
Russula_sp_HM141050_SH1509932.08FU_F_ITS2
Russula_sp_KU141260_SH1509953.08FU_F_ITS2
Russula_sp_MT755627_SH1509964.08FU_F_ITS2
Russula_sp_DQ061922_SH1509939.08FU_F_ITS2
Russula_sp_DQ061921_SH1509943.08FU_F_ITS2
Russula_chloroides_MG687366_SH1509935.08FU_F_ITS2
Russula_chloroides_KX389122_SH1509954.08FU_F_ITS2
Russula_sp_DQ777985_SH1509945.08FU_F_ITS2
Russula_sp_FJ196949_SH1145789.08FU_F_ITS2
Russula_sp_GU205129_SH1509936.08FU_F_ITS2
Russula_sp_JQ347191_SH1509937.08FU_F_ITS2

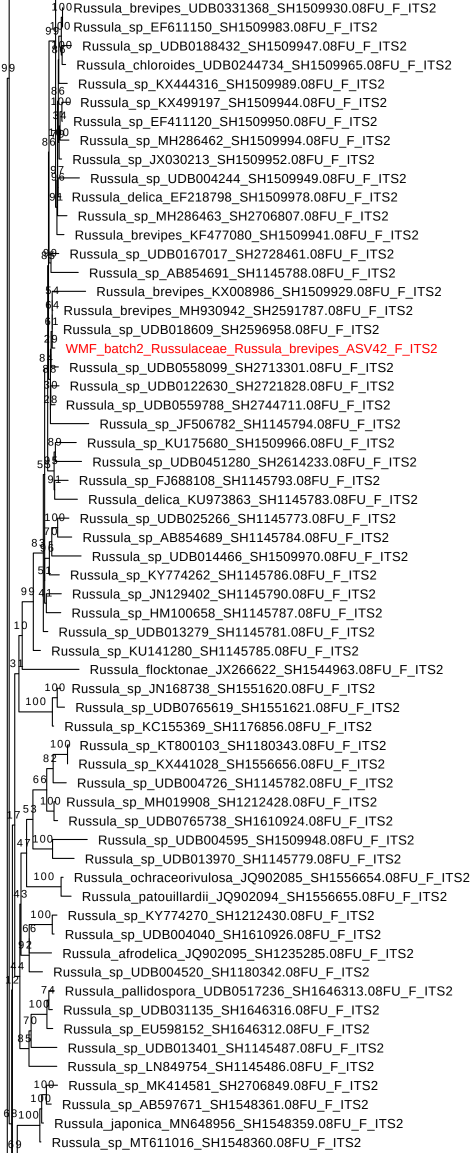

Russula_brevipes_UDB0331368_SH1509930.08FU_F_ITS2
Russula_sp_EF611150_SH1509983.08FU_F_ITS2
Russula_sp_UDB0188432_SH1509947.08FU_F_ITS2
Russula_chloroides_UDB0244734_SH1509965.08FU_F_ITS2
Russula_sp_KX444316_SH1509989.08FU_F_ITS2
Russula_sp_KX499197_SH1509944.08FU_F_ITS2
Russula_sp_EF411120_SH1509950.08FU_F_ITS2
Russula_sp_MH286462_SH1509994.08FU_F_ITS2
Russula_sp_JX030213_SH1509952.08FU_F_ITS2
Russula_sp_UDB004244_SH1509949.08FU_F_ITS2
Russula_delica_EF218798_SH1509978.08FU_F_ITS2
Russula_sp_MH286463_SH2706807.08FU_F_ITS2
Russula_brevipes_KF477080_SH1509941.08FU_F_ITS2
Russula_sp_UDB0167017_SH2728461.08FU_F_ITS2
Russula_sp_AB854691_SH1145788.08FU_F_ITS2
Russula_brevipes_KX008986_SH1509929.08FU_F_ITS2
Russula_brevipes_MH930942_SH2591787.08FU_F_ITS2
Russula_sp_UDB018609_SH2596958.08FU_F_ITS2
WMF_batch2_Russulaceae_Russula_brevipes_ASV42_F_ITS2
Russula_sp_UDB0558099_SH2713301.08FU_F_ITS2
Russula_sp_UDB0122630_SH2721828.08FU_F_ITS2
Russula_sp_UDB0559788_SH2744711.08FU_F_ITS2
Russula_sp_JF506782_SH1145794.08FU_F_ITS2
Russula_sp_KU175680_SH1509966.08FU_F_ITS2
Russula_sp_UDB0451280_SH2614233.08FU_F_ITS2
Russula_sp_FJ688108_SH1145793.08FU_F_ITS2
Russula_delica_KU973863_SH1145783.08FU_F_ITS2
Russula_sp_UDB025266_SH1145773.08FU_F_ITS2
Russula_sp_AB854689_SH1145784.08FU_F_ITS2
Russula_sp_UDB014466_SH1509970.08FU_F_ITS2
Russula_sp_KY774262_SH1145786.08FU_F_ITS2
Russula_sp_JN129402_SH1145790.08FU_F_ITS2
Russula_sp_HM100658_SH1145787.08FU_F_ITS2
Russula_sp_UDB013279_SH1145781.08FU_F_ITS2
Russula_sp_KU141280_SH1145785.08FU_F_ITS2
Russula_flocktonae_JX266622_SH1544963.08FU_F_ITS2
Russula_sp_JN168738_SH1551620.08FU_F_ITS2
Russula_sp_UDB0765619_SH1551621.08FU_F_ITS2
Russula_sp_KC155369_SH1176856.08FU_F_ITS2
Russula_sp_KT800103_SH1180343.08FU_F_ITS2
Russula_sp_KX441028_SH1556656.08FU_F_ITS2
Russula_sp_UDB004726_SH1145782.08FU_F_ITS2
Russula_sp_MH019908_SH1212428.08FU_F_ITS2
Russula_sp_UDB0765738_SH1610924.08FU_F_ITS2
Russula_sp_UDB004595_SH1509948.08FU_F_ITS2
Russula_sp_UDB013970_SH1145779.08FU_F_ITS2
Russula_ochraceorivulosa_JQ902085_SH1556654.08FU_F_ITS2
Russula_patouillardii_JQ902094_SH1556655.08FU_F_ITS2
Russula_sp_KY774270_SH1212430.08FU_F_ITS2
Russula_sp_UDB004040_SH1610926.08FU_F_ITS2
Russula_afrodelica_JQ902095_SH1235285.08FU_F_ITS2
Russula_sp_UDB004520_SH1180342.08FU_F_ITS2
Russula_pallidospora_UDB0517236_SH1646313.08FU_F_ITS2
Russula_sp_UDB031135_SH1646316.08FU_F_ITS2
Russula_sp_EU598152_SH1646312.08FU_F_ITS2
Russula_sp_UDB013401_SH1145487.08FU_F_ITS2
Russula_sp_LN849754_SH1145486.08FU_F_ITS2
Russula_sp_MK414581_SH2706849.08FU_F_ITS2
Russula_sp_AB597671_SH1548361.08FU_F_ITS2
Russula_japonica_MN648956_SH1548359.08FU_F_ITS2
Russula_sp_MT611016_SH1548360.08FU_F_ITS2

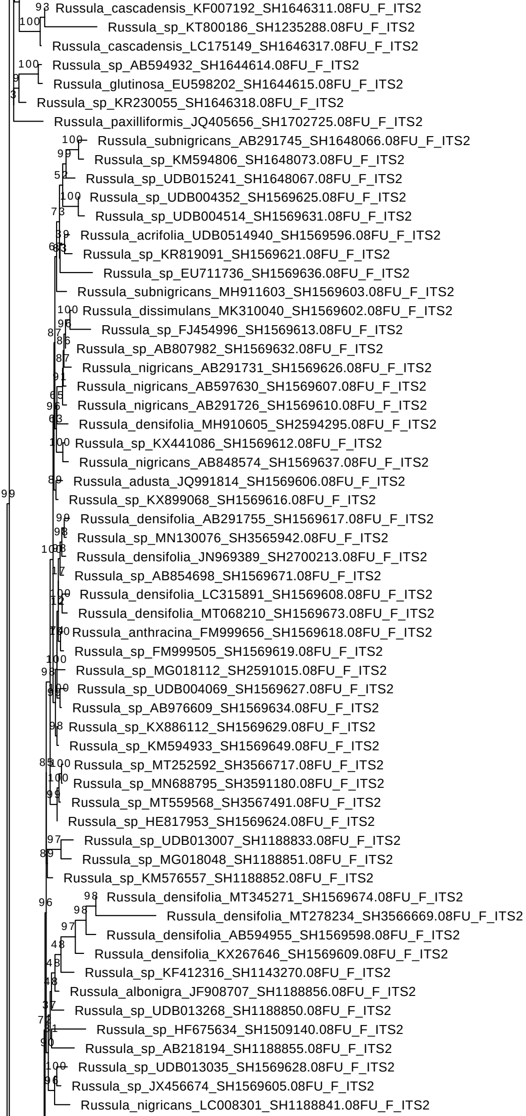
Russula_cascadensis_KF007192_SH1646311.08FU_F_ITS2
Russula_sp_KT800186_SH1235288.08FU_F_ITS2
Russula_cascadensis_LC175149_SH1646317.08FU_F_ITS2
Russula_sp_AB594932_SH1644614.08FU_F_ITS2
Russula_glutinosa_EU598202_SH1644615.08FU_F_ITS2
Russula_sp_KR230055_SH1646318.08FU_F_ITS2
Russula_paxilliformis_JQ405656_SH1702725.08FU_F_ITS2
Russula_subnigricans_AB291745_SH1648066.08FU_F_ITS2
Russula_sp_KM594806_SH1648073.08FU_F_ITS2
Russula_sp_UDB015241_SH1648067.08FU_F_ITS2
Russula_sp_UDB004352_SH1569625.08FU_F_ITS2
Russula_sp_UDB004514_SH1569631.08FU_F_ITS2
Russula_acrifolia_UDB0514940_SH1569596.08FU_F_ITS2
Russula_sp_KR819091_SH1569621.08FU_F_ITS2
Russula_sp_EU711736_SH1569636.08FU_F_ITS2
Russula_subnigricans_MH911603_SH1569603.08FU_F_ITS2
Russula_dissimulans_MK310040_SH1569602.08FU_F_ITS2
Russula_sp_FJ454996_SH1569613.08FU_F_ITS2
Russula_sp_AB807982_SH1569632.08FU_F_ITS2
Russula_nigricans_AB291731_SH1569626.08FU_F_ITS2
Russula_nigricans_AB597630_SH1569607.08FU_F_ITS2
Russula_nigricans_AB291726_SH1569610.08FU_F_ITS2
Russula_densifolia_MH910605_SH2594295.08FU_F_ITS2
Russula_sp_KX441086_SH1569612.08FU_F_ITS2
Russula_nigricans_AB848574_SH1569637.08FU_F_ITS2
Russula_adusta_JQ991814_SH1569606.08FU_F_ITS2
Russula_sp_KX899068_SH1569616.08FU_F_ITS2
Russula_densifolia_AB291755_SH1569617.08FU_F_ITS2
Russula_sp_MN130076_SH3565942.08FU_F_ITS2
Russula_densifolia_JN969389_SH2700213.08FU_F_ITS2
Russula_sp_AB854698_SH1569671.08FU_F_ITS2
Russula_densifolia_LC315891_SH1569608.08FU_F_ITS2
Russula_densifolia_MT068210_SH1569673.08FU_F_ITS2
Russula_anthracina_FM999656_SH1569618.08FU_F_ITS2
Russula_sp_FM999505_SH1569619.08FU_F_ITS2
Russula_sp_MG018112_SH2591015.08FU_F_ITS2
Russula_sp_UDB004069_SH1569627.08FU_F_ITS2
Russula_sp_AB976609_SH1569634.08FU_F_ITS2
Russula_sp_KX886112_SH1569629.08FU_F_ITS2
Russula_sp_KM594933_SH1569649.08FU_F_ITS2
Russula_sp_MT252592_SH3566717.08FU_F_ITS2
Russula_sp_MN688795_SH3591180.08FU_F_ITS2
Russula_sp_MT559568_SH3567491.08FU_F_ITS2
Russula_sp_HE817953_SH1569624.08FU_F_ITS2
Russula_sp_UDB013007_SH1188833.08FU_F_ITS2
Russula_sp_MG018048_SH1188851.08FU_F_ITS2
Russula_sp_KM576557_SH1188852.08FU_F_ITS2
Russula_densifolia_MT345271_SH1569674.08FU_F_ITS2
Russula_densifolia_MT278234_SH3566669.08FU_F_ITS2
Russula_densifolia_AB594955_SH1569598.08FU_F_ITS2
Russula_densifolia_KX267646_SH1569609.08FU_F_ITS2
Russula_sp_KF412316_SH1143270.08FU_F_ITS2
Russula_albonigra_JF908707_SH1188856.08FU_F_ITS2
Russula_sp_UDB013268_SH1188850.08FU_F_ITS2
Russula_sp_HF675634_SH1509140.08FU_F_ITS2
Russula_sp_AB218194_SH1188855.08FU_F_ITS2
Russula_sp_UDB013035_SH1569628.08FU_F_ITS2
Russula_sp_JX456674_SH1569605.08FU_F_ITS2
Russula_nigricans_LC008301_SH1188841.08FU_F_ITS2

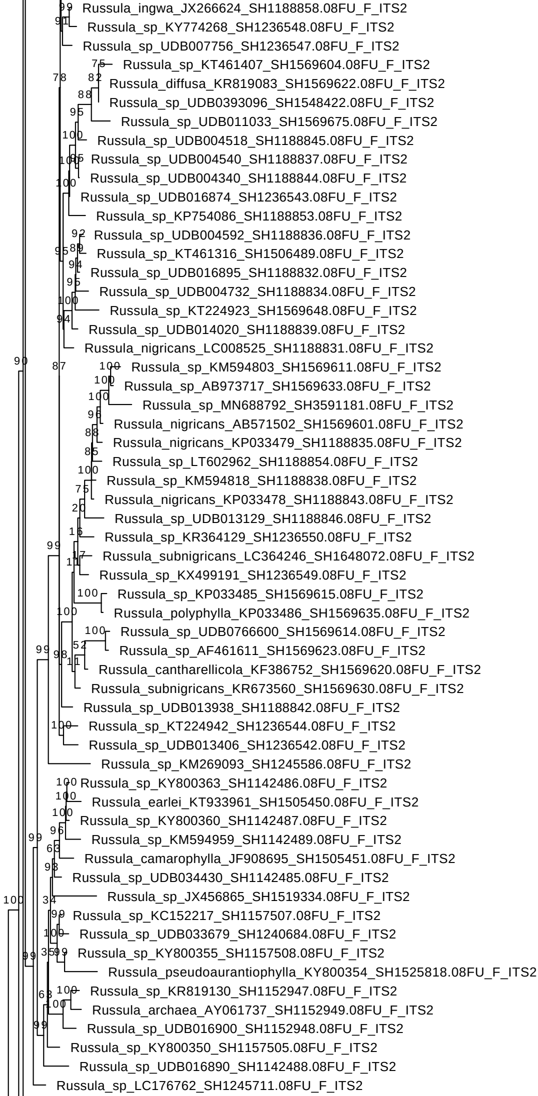
Russula_ingwa_JX266624_SH1188858.08FU_F_ITS2
Russula_sp_KY774268_SH1236548.08FU_F_ITS2
Russula_sp_UDB007756_SH1236547.08FU_F_ITS2
Russula_sp_KT461407_SH1569604.08FU_F_ITS2
Russula_diffusa_KR819083_SH1569622.08FU_F_ITS2
Russula_sp_UDB0393096_SH1548422.08FU_F_ITS2
Russula_sp_UDB011033_SH1569675.08FU_F_ITS2
Russula_sp_UDB004518_SH1188845.08FU_F_ITS2
Russula_sp_UDB004540_SH1188837.08FU_F_ITS2
Russula_sp_UDB004340_SH1188844.08FU_F_ITS2
Russula_sp_UDB016874_SH1236543.08FU_F_ITS2
Russula_sp_KP754086_SH1188853.08FU_F_ITS2
Russula_sp_UDB004592_SH1188836.08FU_F_ITS2
Russula_sp_KT461316_SH1506489.08FU_F_ITS2
Russula_sp_UDB016895_SH1188832.08FU_F_ITS2
Russula_sp_UDB004732_SH1188834.08FU_F_ITS2
Russula_sp_KT224923_SH1569648.08FU_F_ITS2
Russula_sp_UDB014020_SH1188839.08FU_F_ITS2
Russula_nigricans_LC008525_SH1188831.08FU_F_ITS2
Russula_sp_KM594803_SH1569611.08FU_F_ITS2
Russula_sp_AB973717_SH1569633.08FU_F_ITS2
Russula_sp_MN688792_SH3591181.08FU_F_ITS2
Russula_nigricans_AB571502_SH1569601.08FU_F_ITS2
Russula_nigricans_KP033479_SH1188835.08FU_F_ITS2
Russula_sp_LT602962_SH1188854.08FU_F_ITS2
Russula_sp_KM594818_SH1188838.08FU_F_ITS2
Russula_nigricans_KP033478_SH1188843.08FU_F_ITS2
Russula_sp_UDB013129_SH1188846.08FU_F_ITS2
Russula_sp_KR364129_SH1236550.08FU_F_ITS2
Russula_subnigricans_LC364246_SH1648072.08FU_F_ITS2
Russula_sp_KX499191_SH1236549.08FU_F_ITS2
Russula_sp_KP033485_SH1569615.08FU_F_ITS2
Russula_polyphylla_KP033486_SH1569635.08FU_F_ITS2
Russula_sp_UDB0766600_SH1569614.08FU_F_ITS2
Russula_sp_AF461611_SH1569623.08FU_F_ITS2
Russula_cantharellicola_KF386752_SH1569620.08FU_F_ITS2
Russula_subnigricans_KR673560_SH1569630.08FU_F_ITS2
Russula_sp_UDB013938_SH1188842.08FU_F_ITS2
Russula_sp_KT224942_SH1236544.08FU_F_ITS2
Russula_sp_UDB013406_SH1236542.08FU_F_ITS2
Russula_sp_KM269093_SH1245586.08FU_F_ITS2
Russula_sp_KY800363_SH1142486.08FU_F_ITS2
Russula_earlei_KT933961_SH1505450.08FU_F_ITS2
Russula_sp_KY800360_SH1142487.08FU_F_ITS2
Russula_sp_KM594959_SH1142489.08FU_F_ITS2
Russula_camarophylla_JF908695_SH1505451.08FU_F_ITS2
Russula_sp_UDB034430_SH1142485.08FU_F_ITS2
Russula_sp_JX456865_SH1519334.08FU_F_ITS2
Russula_sp_KC152217_SH1157507.08FU_F_ITS2
Russula_sp_UDB033679_SH1240684.08FU_F_ITS2
Russula_sp_KY800355_SH1157508.08FU_F_ITS2
Russula_pseudoaurantiophylla_KY800354_SH1525818.08FU_F_ITS2
Russula_sp_KR819130_SH1152947.08FU_F_ITS2
Russula_archaea_AY061737_SH1152949.08FU_F_ITS2
Russula_sp_UDB016900_SH1152948.08FU_F_ITS2
Russula_sp_KY800350_SH1157505.08FU_F_ITS2
Russula_sp_UDB016890_SH1142488.08FU_F_ITS2
Russula_sp_LC176762_SH1245711.08FU_F_ITS2

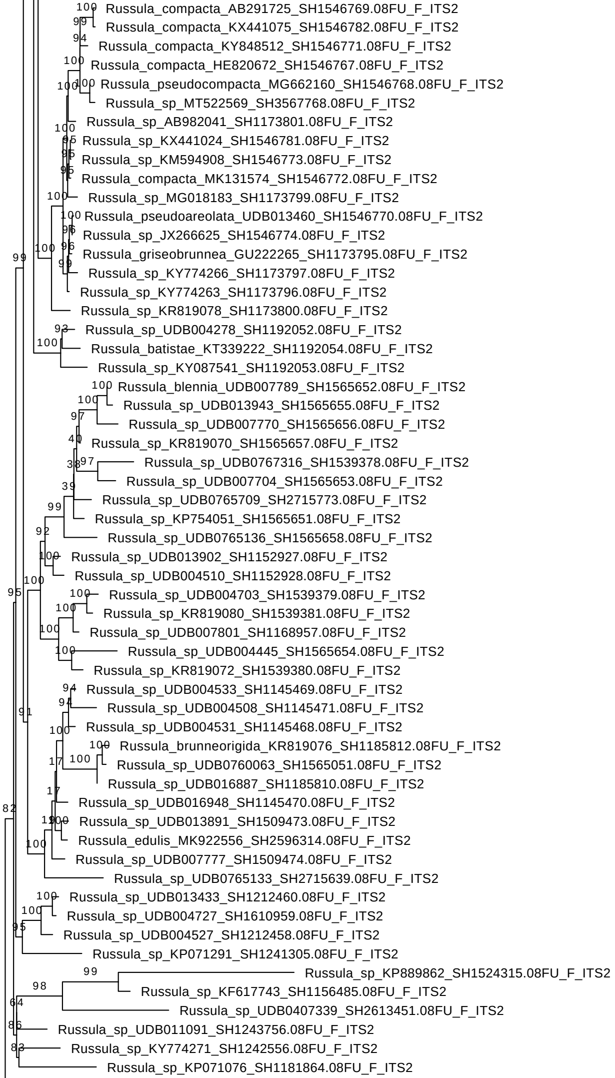
Russula_compacta_AB291725_SH1546769.08FU_F_ITS2
Russula_compacta_KX441075_SH1546782.08FU_F_ITS2
Russula_compacta_KY848512_SH1546771.08FU_F_ITS2
Russula_compacta_HE820672_SH1546767.08FU_F_ITS2
Russula_pseudocompacta_MG662160_SH1546768.08FU_F_ITS2
Russula_sp_MT522569_SH3567768.08FU_F_ITS2
Russula_sp_AB982041_SH1173801.08FU_F_ITS2
Russula_sp_KX441024_SH1546781.08FU_F_ITS2
Russula_sp_KM594908_SH1546773.08FU_F_ITS2
Russula_compacta_MK131574_SH1546772.08FU_F_ITS2
Russula_sp_MG018183_SH1173799.08FU_F_ITS2
Russula_pseudoareolata_UDB013460_SH1546770.08FU_F_ITS2
Russula_sp_JX266625_SH1546774.08FU_F_ITS2
Russula_griseobrunnea_GU222265_SH1173795.08FU_F_ITS2
Russula_sp_KY774266_SH1173797.08FU_F_ITS2
Russula_sp_KY774263_SH1173796.08FU_F_ITS2
Russula_sp_KR819078_SH1173800.08FU_F_ITS2
Russula_sp_UDB004278_SH1192052.08FU_F_ITS2
Russula_batistae_KT339222_SH1192054.08FU_F_ITS2
Russula_sp_KY087541_SH1192053.08FU_F_ITS2
Russula_blennia_UDB007789_SH1565652.08FU_F_ITS2
Russula_sp_UDB013943_SH1565655.08FU_F_ITS2
Russula_sp_UDB007770_SH1565656.08FU_F_ITS2
Russula_sp_KR819070_SH1565657.08FU_F_ITS2
Russula_sp_UDB0767316_SH1539378.08FU_F_ITS2
Russula_sp_UDB007704_SH1565653.08FU_F_ITS2
Russula_sp_UDB0765709_SH2715773.08FU_F_ITS2
Russula_sp_KP754051_SH1565651.08FU_F_ITS2
Russula_sp_UDB0765136_SH1565658.08FU_F_ITS2
Russula_sp_UDB013902_SH1152927.08FU_F_ITS2
Russula_sp_UDB004510_SH1152928.08FU_F_ITS2
Russula_sp_UDB004703_SH1539379.08FU_F_ITS2
Russula_sp_KR819080_SH1539381.08FU_F_ITS2
Russula_sp_UDB007801_SH1168957.08FU_F_ITS2
Russula_sp_UDB004445_SH1565654.08FU_F_ITS2
Russula_sp_KR819072_SH1539380.08FU_F_ITS2
Russula_sp_UDB004533_SH1145469.08FU_F_ITS2
Russula_sp_UDB004508_SH1145471.08FU_F_ITS2
Russula_sp_UDB004531_SH1145468.08FU_F_ITS2
Russula_brunneorigida_KR819076_SH1185812.08FU_F_ITS2
Russula_sp_UDB0760063_SH1565051.08FU_F_ITS2
Russula_sp_UDB016887_SH1185810.08FU_F_ITS2
Russula_sp_UDB016948_SH1145470.08FU_F_ITS2
Russula_sp_UDB013891_SH1509473.08FU_F_ITS2
Russula_edulis_MK922556_SH2596314.08FU_F_ITS2
Russula_sp_UDB007777_SH1509474.08FU_F_ITS2
Russula_sp_UDB0765133_SH2715639.08FU_F_ITS2
Russula_sp_UDB013433_SH1212460.08FU_F_ITS2
Russula_sp_UDB004727_SH1610959.08FU_F_ITS2
Russula_sp_UDB004527_SH1212458.08FU_F_ITS2
Russula_sp_KP071291_SH1241305.08FU_F_ITS2
Russula_sp_KP889862_SH1524315.08FU_F_ITS2
Russula_sp_KF617743_SH1156485.08FU_F_ITS2
Russula_sp_UDB0407339_SH2613451.08FU_F_ITS2
Russula_sp_UDB011091_SH1243756.08FU_F_ITS2
Russula_sp_KY774271_SH1242556.08FU_F_ITS2
Russula_sp_KP071076_SH1181864.08FU_F_ITS2

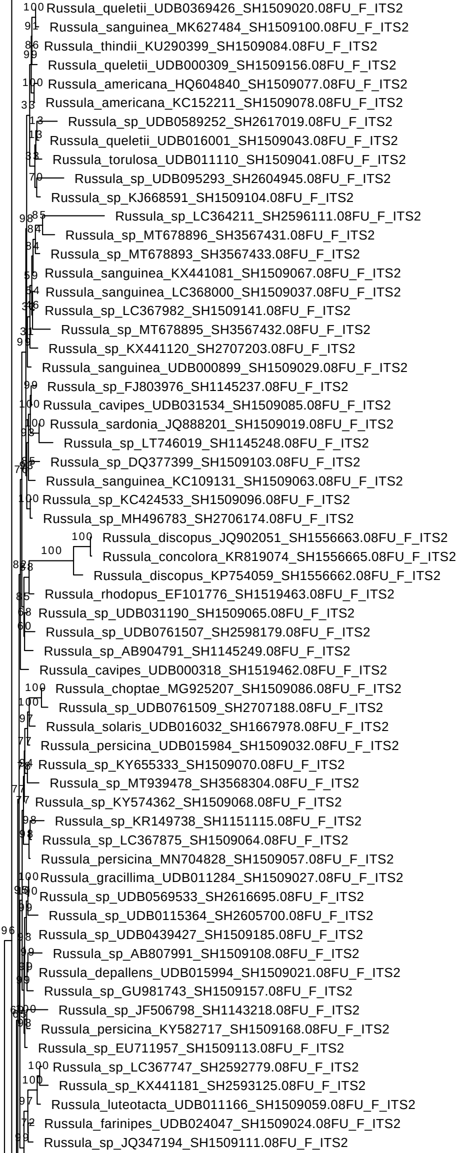
Russula_queletii_UDB0369426_SH1509020.08FU_F_ITS2
Russula_sanguinea_MK627484_SH1509100.08FU_F_ITS2
Russula_thindii_KU290399_SH1509084.08FU_F_ITS2
Russula_queletii_UDB000309_SH1509156.08FU_F_ITS2
Russula_americana_HQ604840_SH1509077.08FU_F_ITS2
Russula_americana_KC152211_SH1509078.08FU_F_ITS2
Russula_sp_UDB0589252_SH2617019.08FU_F_ITS2
Russula_queletii_UDB016001_SH1509043.08FU_F_ITS2
Russula_torulosa_UDB011110_SH1509041.08FU_F_ITS2
Russula_sp_UDB095293_SH2604945.08FU_F_ITS2
Russula_sp_KJ668591_SH1509104.08FU_F_ITS2
Russula_sp_LC364211_SH2596111.08FU_F_ITS2
Russula_sp_MT678896_SH3567431.08FU_F_ITS2
Russula_sp_MT678893_SH3567433.08FU_F_ITS2
Russula_sanguinea_KX441081_SH1509067.08FU_F_ITS2
Russula_sanguinea_LC368000_SH1509037.08FU_F_ITS2
Russula_sp_LC367982_SH1509141.08FU_F_ITS2
Russula_sp_MT678895_SH3567432.08FU_F_ITS2
Russula_sp_KX441120_SH2707203.08FU_F_ITS2
Russula_sanguinea_UDB000899_SH1509029.08FU_F_ITS2
Russula_sp_FJ803976_SH1145237.08FU_F_ITS2
Russula_cavipes_UDB031534_SH1509085.08FU_F_ITS2
Russula_sardonia_JQ888201_SH1509019.08FU_F_ITS2
Russula_sp_LT746019_SH1145248.08FU_F_ITS2
Russula_sp_DQ377399_SH1509103.08FU_F_ITS2
Russula_sanguinea_KC109131_SH1509063.08FU_F_ITS2
Russula_sp_KC424533_SH1509096.08FU_F_ITS2
Russula_sp_MH496783_SH2706174.08FU_F_ITS2
Russula_discopus_JQ902051_SH1556663.08FU_F_ITS2
Russula_concolora_KR819074_SH1556665.08FU_F_ITS2
Russula_discopus_KP754059_SH1556662.08FU_F_ITS2
Russula_rhodopus_EF101776_SH1519463.08FU_F_ITS2
Russula_sp_UDB031190_SH1509065.08FU_F_ITS2
Russula_sp_UDB0761507_SH2598179.08FU_F_ITS2
Russula_sp_AB904791_SH1145249.08FU_F_ITS2
Russula_cavipes_UDB000318_SH1519462.08FU_F_ITS2
Russula_choptae_MG925207_SH1509086.08FU_F_ITS2
Russula_sp_UDB0761509_SH2707188.08FU_F_ITS2
Russula_solaris_UDB016032_SH1667978.08FU_F_ITS2
Russula_persicina_UDB015984_SH1509032.08FU_F_ITS2
Russula_sp_KY655333_SH1509070.08FU_F_ITS2
Russula_sp_MT939478_SH3568304.08FU_F_ITS2
Russula_sp_KY574362_SH1509068.08FU_F_ITS2
Russula_sp_KR149738_SH1151115.08FU_F_ITS2
Russula_sp_LC367875_SH1509064.08FU_F_ITS2
Russula_persicina_MN704828_SH1509057.08FU_F_ITS2
Russula_gracillima_UDB011284_SH1509027.08FU_F_ITS2
Russula_sp_UDB0569533_SH2616695.08FU_F_ITS2
Russula_sp_UDB0115364_SH2605700.08FU_F_ITS2
Russula_sp_UDB0439427_SH1509185.08FU_F_ITS2
Russula_sp_AB807991_SH1509108.08FU_F_ITS2
Russula_depallens_UDB015994_SH1509021.08FU_F_ITS2
Russula_sp_GU981743_SH1509157.08FU_F_ITS2
Russula_sp_JF506798_SH1143218.08FU_F_ITS2
Russula_persicina_KY582717_SH1509168.08FU_F_ITS2
Russula_sp_EU711957_SH1509113.08FU_F_ITS2
Russula_sp_LC367747_SH2592779.08FU_F_ITS2
Russula_sp_KX441181_SH2593125.08FU_F_ITS2
Russula_luteotacta_UDB011166_SH1509059.08FU_F_ITS2
Russula_farinipes_UDB024047_SH1509024.08FU_F_ITS2
Russula_sp_JQ347194_SH1509111.08FU_F_ITS2

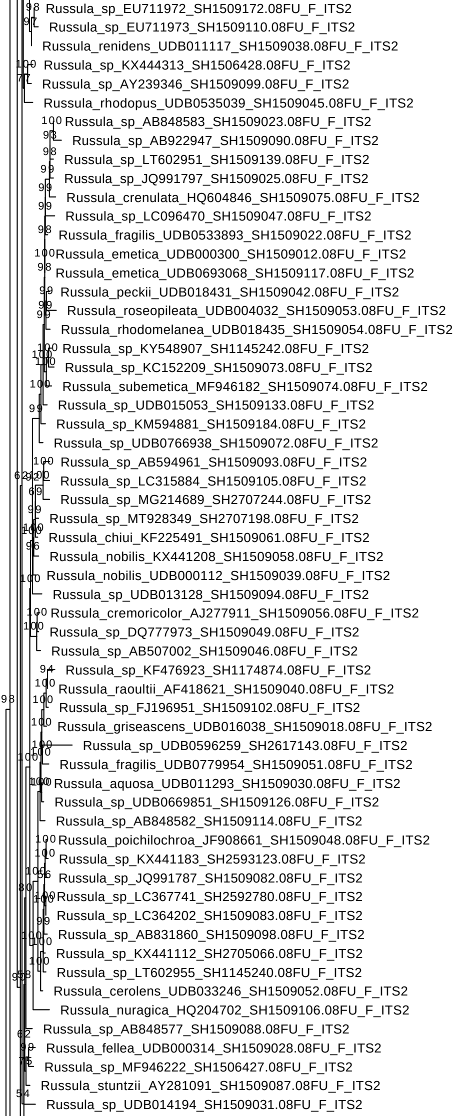
Russula_sp_EU711972_SH1509172.08FU_F_ITS2
Russula_sp_EU711973_SH1509110.08FU_F_ITS2
Russula_renidens_UDB011117_SH1509038.08FU_F_ITS2
Russula_sp_KX444313_SH1506428.08FU_F_ITS2
Russula_sp_AY239346_SH1509099.08FU_F_ITS2
Russula_rhodopus_UDB0535039_SH1509045.08FU_F_ITS2
Russula_sp_AB848583_SH1509023.08FU_F_ITS2
Russula_sp_AB922947_SH1509090.08FU_F_ITS2
Russula_sp_LT602951_SH1509139.08FU_F_ITS2
Russula_sp_JQ991797_SH1509025.08FU_F_ITS2
Russula_crenulata_HQ604846_SH1509075.08FU_F_ITS2
Russula_sp_LC096470_SH1509047.08FU_F_ITS2
Russula_fragilis_UDB0533893_SH1509022.08FU_F_ITS2
Russula_emetica_UDB000300_SH1509012.08FU_F_ITS2
Russula_emetica_UDB0693068_SH1509117.08FU_F_ITS2
Russula_peckii_UDB018431_SH1509042.08FU_F_ITS2
Russula_roseopileata_UDB004032_SH1509053.08FU_F_ITS2
Russula_rhodomelanea_UDB018435_SH1509054.08FU_F_ITS2
Russula_sp_KY548907_SH1145242.08FU_F_ITS2
Russula_sp_KC152209_SH1509073.08FU_F_ITS2
Russula_subemetica_MF946182_SH1509074.08FU_F_ITS2
Russula_sp_UDB015053_SH1509133.08FU_F_ITS2
Russula_sp_KM594881_SH1509184.08FU_F_ITS2
Russula_sp_UDB0766938_SH1509072.08FU_F_ITS2
Russula_sp_AB594961_SH1509093.08FU_F_ITS2
Russula_sp_LC315884_SH1509105.08FU_F_ITS2
Russula_sp_MG214689_SH2707244.08FU_F_ITS2
Russula_sp_MT928349_SH2707198.08FU_F_ITS2
Russula_chiui_KF225491_SH1509061.08FU_F_ITS2
Russula_nobilis_KX441208_SH1509058.08FU_F_ITS2
Russula_nobilis_UDB000112_SH1509039.08FU_F_ITS2
Russula_sp_UDB013128_SH1509094.08FU_F_ITS2
Russula_cremoricolor_AJ277911_SH1509056.08FU_F_ITS2
Russula_sp_DQ777973_SH1509049.08FU_F_ITS2
Russula_sp_AB507002_SH1509046.08FU_F_ITS2
Russula_sp_KF476923_SH1174874.08FU_F_ITS2
Russula_raoultii_AF418621_SH1509040.08FU_F_ITS2
Russula_sp_FJ196951_SH1509102.08FU_F_ITS2
Russula_griseascens_UDB016038_SH1509018.08FU_F_ITS2
Russula_sp_UDB0596259_SH2617143.08FU_F_ITS2
Russula_fragilis_UDB0779954_SH1509051.08FU_F_ITS2
Russula_aquosa_UDB011293_SH1509030.08FU_F_ITS2
Russula_sp_UDB0669851_SH1509126.08FU_F_ITS2
Russula_sp_AB848582_SH1509114.08FU_F_ITS2
Russula_poichilochroa_JF908661_SH1509048.08FU_F_ITS2
Russula_sp_KX441183_SH2593123.08FU_F_ITS2
Russula_sp_JQ991787_SH1509082.08FU_F_ITS2
Russula_sp_LC367741_SH2592780.08FU_F_ITS2
Russula_sp_LC364202_SH1509083.08FU_F_ITS2
Russula_sp_AB831860_SH1509098.08FU_F_ITS2
Russula_sp_KX441112_SH2705066.08FU_F_ITS2
Russula_sp_LT602955_SH1145240.08FU_F_ITS2
Russula_cerolens_UDB033246_SH1509052.08FU_F_ITS2
Russula_nuragica_HQ204702_SH1509106.08FU_F_ITS2
Russula_sp_AB848577_SH1509088.08FU_F_ITS2
Russula_fellea_UDB000314_SH1509028.08FU_F_ITS2
Russula_sp_MF946222_SH1506427.08FU_F_ITS2
Russula_stuntzii_AY281091_SH1509087.08FU_F_ITS2
Russula_sp_UDB014194_SH1509031.08FU_F_ITS2

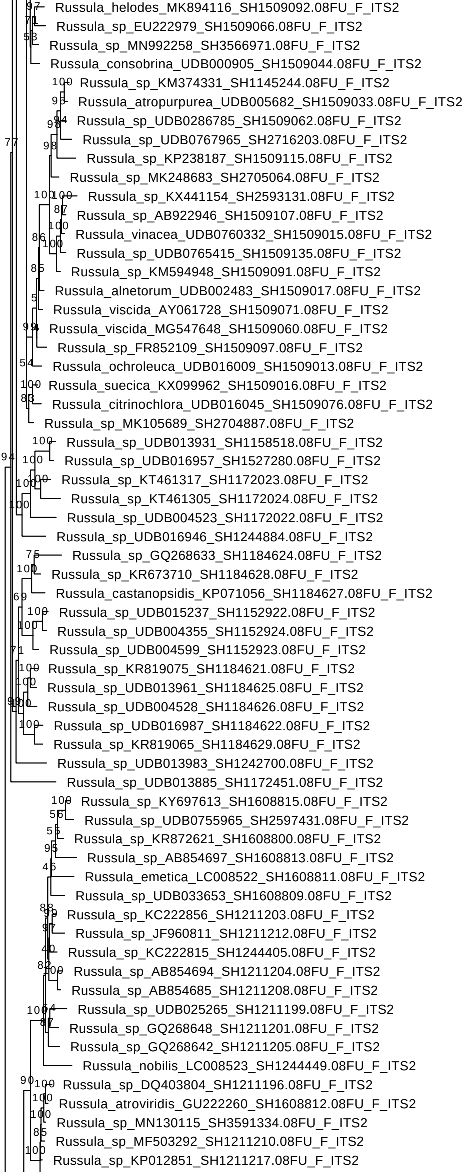
Russula_helodes_MK894116_SH1509092.08FU_F_ITS2
Russula_sp_EU222979_SH1509066.08FU_F_ITS2
Russula_sp_MN992258_SH3566971.08FU_F_ITS2
Russula_consobrina_UDB000905_SH1509044.08FU_F_ITS2
Russula_sp_KM374331_SH1145244.08FU_F_ITS2
Russula_atropurpurea_UDB005682_SH1509033.08FU_F_ITS2
Russula_sp_UDB0286785_SH1509062.08FU_F_ITS2
Russula_sp_UDB0767965_SH2716203.08FU_F_ITS2
Russula_sp_KP238187_SH1509115.08FU_F_ITS2
Russula_sp_MK248683_SH2705064.08FU_F_ITS2
Russula_sp_KX441154_SH2593131.08FU_F_ITS2
Russula_sp_AB922946_SH1509107.08FU_F_ITS2
Russula_vinacea_UDB0760332_SH1509015.08FU_F_ITS2
Russula_sp_UDB0765415_SH1509135.08FU_F_ITS2
Russula_sp_KM594948_SH1509091.08FU_F_ITS2
Russula_alnetorum_UDB002483_SH1509017.08FU_F_ITS2
Russula_viscida_AY061728_SH1509071.08FU_F_ITS2
Russula_viscida_MG547648_SH1509060.08FU_F_ITS2
Russula_sp_FR852109_SH1509097.08FU_F_ITS2
Russula_ochroleuca_UDB016009_SH1509013.08FU_F_ITS2
Russula_suecica_KX099962_SH1509016.08FU_F_ITS2
Russula_citrinochlora_UDB016045_SH1509076.08FU_F_ITS2
Russula_sp_MK105689_SH2704887.08FU_F_ITS2
Russula_sp_UDB013931_SH1158518.08FU_F_ITS2
Russula_sp_UDB016957_SH1527280.08FU_F_ITS2
Russula_sp_KT461317_SH1172023.08FU_F_ITS2
Russula_sp_KT461305_SH1172024.08FU_F_ITS2
Russula_sp_UDB004523_SH1172022.08FU_F_ITS2
Russula_sp_UDB016946_SH1244884.08FU_F_ITS2
Russula_sp_GQ268633_SH1184624.08FU_F_ITS2
Russula_sp_KR673710_SH1184628.08FU_F_ITS2
Russula_castanopsidis_KP071056_SH1184627.08FU_F_ITS2
Russula_sp_UDB015237_SH1152922.08FU_F_ITS2
Russula_sp_UDB004355_SH1152924.08FU_F_ITS2
Russula_sp_UDB004599_SH1152923.08FU_F_ITS2
Russula_sp_KR819075_SH1184621.08FU_F_ITS2
Russula_sp_UDB013961_SH1184625.08FU_F_ITS2
Russula_sp_UDB004528_SH1184626.08FU_F_ITS2
Russula_sp_UDB016987_SH1184622.08FU_F_ITS2
Russula_sp_KR819065_SH1184629.08FU_F_ITS2
Russula_sp_UDB013983_SH1242700.08FU_F_ITS2
Russula_sp_UDB013885_SH1172451.08FU_F_ITS2
Russula_sp_KY697613_SH1608815.08FU_F_ITS2
Russula_sp_UDB0755965_SH2597431.08FU_F_ITS2
Russula_sp_KR872621_SH1608800.08FU_F_ITS2
Russula_sp_AB854697_SH1608813.08FU_F_ITS2
Russula_emetica_LC008522_SH1608811.08FU_F_ITS2
Russula_sp_UDB033653_SH1608809.08FU_F_ITS2
Russula_sp_KC222856_SH1211203.08FU_F_ITS2
Russula_sp_JF960811_SH1211212.08FU_F_ITS2
Russula_sp_KC222815_SH1244405.08FU_F_ITS2
Russula_sp_AB854694_SH1211204.08FU_F_ITS2
Russula_sp_AB854685_SH1211208.08FU_F_ITS2
Russula_sp_UDB025265_SH1211199.08FU_F_ITS2
Russula_sp_GQ268648_SH1211201.08FU_F_ITS2
Russula_sp_GQ268642_SH1211205.08FU_F_ITS2
Russula_nobilis_LC008523_SH1244449.08FU_F_ITS2
Russula_sp_DQ403804_SH1211196.08FU_F_ITS2
Russula_atroviridis_GU222260_SH1608812.08FU_F_ITS2
Russula_sp_MN130115_SH3591334.08FU_F_ITS2
Russula_sp_MF503292_SH1211210.08FU_F_ITS2
Russula_sp_KP012851_SH1211217.08FU_F_ITS2

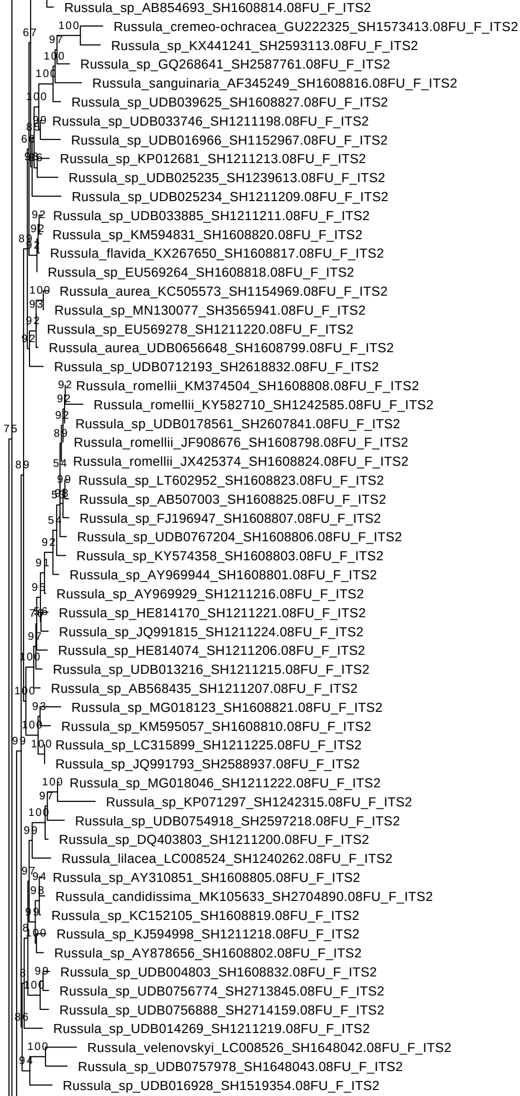
Russula_sp_AB854693_SH1608814.08FU_F_ITS2
Russula_cremeo-ochracea_GU222325_SH1573413.08FU_F_ITS2
Russula_sp_KX441241_SH2593113.08FU_F_ITS2
Russula_sp_GQ268641_SH2587761.08FU_F_ITS2
Russula_sanguinaria_AF345249_SH1608816.08FU_F_ITS2
Russula_sp_UDB039625_SH1608827.08FU_F_ITS2
Russula_sp_UDB033746_SH1211198.08FU_F_ITS2
Russula_sp_UDB016966_SH1152967.08FU_F_ITS2
Russula_sp_KP012681_SH1211213.08FU_F_ITS2
Russula_sp_UDB025235_SH1239613.08FU_F_ITS2
Russula_sp_UDB025234_SH1211209.08FU_F_ITS2
Russula_sp_UDB033885_SH1211211.08FU_F_ITS2
Russula_sp_KM594831_SH1608820.08FU_F_ITS2
Russula_flavida_KX267650_SH1608817.08FU_F_ITS2
Russula_sp_EU569264_SH1608818.08FU_F_ITS2
Russula_aurea_KC505573_SH1154969.08FU_F_ITS2
Russula_sp_MN130077_SH3565941.08FU_F_ITS2
Russula_sp_EU569278_SH1211220.08FU_F_ITS2
Russula_aurea_UDB0656648_SH1608799.08FU_F_ITS2
Russula_sp_UDB0712193_SH2618832.08FU_F_ITS2
Russula_romellii_KM374504_SH1608808.08FU_F_ITS2
Russula_romellii_KY582710_SH1242585.08FU_F_ITS2
Russula_sp_UDB0178561_SH2607841.08FU_F_ITS2
Russula_romellii_JF908676_SH1608798.08FU_F_ITS2
Russula_romellii_JX425374_SH1608824.08FU_F_ITS2
Russula_sp_LT602952_SH1608823.08FU_F_ITS2
Russula_sp_AB507003_SH1608825.08FU_F_ITS2
Russula_sp_FJ196947_SH1608807.08FU_F_ITS2
Russula_sp_UDB0767204_SH1608806.08FU_F_ITS2
Russula_sp_KY574358_SH1608803.08FU_F_ITS2
Russula_sp_AY969944_SH1608801.08FU_F_ITS2
Russula_sp_AY969929_SH1211216.08FU_F_ITS2
Russula_sp_HE814170_SH1211221.08FU_F_ITS2
Russula_sp_JQ991815_SH1211224.08FU_F_ITS2
Russula_sp_HE814074_SH1211206.08FU_F_ITS2
Russula_sp_UDB013216_SH1211215.08FU_F_ITS2
Russula_sp_AB568435_SH1211207.08FU_F_ITS2
Russula_sp_MG018123_SH1608821.08FU_F_ITS2
Russula_sp_KM595057_SH1608810.08FU_F_ITS2
Russula_sp_LC315899_SH1211225.08FU_F_ITS2
Russula_sp_JQ991793_SH2588937.08FU_F_ITS2
Russula_sp_MG018046_SH1211222.08FU_F_ITS2
Russula_sp_KP071297_SH1242315.08FU_F_ITS2
Russula_sp_UDB0754918_SH2597218.08FU_F_ITS2
Russula_sp_DQ403803_SH1211200.08FU_F_ITS2
Russula_lilacea_LC008524_SH1240262.08FU_F_ITS2
Russula_sp_AY310851_SH1608805.08FU_F_ITS2
Russula_candidissima_MK105633_SH2704890.08FU_F_ITS2
Russula_sp_KC152105_SH1608819.08FU_F_ITS2
Russula_sp_KJ594998_SH1211218.08FU_F_ITS2
Russula_sp_AY878656_SH1608802.08FU_F_ITS2
Russula_sp_UDB004803_SH1608832.08FU_F_ITS2
Russula_sp_UDB0756774_SH2713845.08FU_F_ITS2
Russula_sp_UDB0756888_SH2714159.08FU_F_ITS2
Russula_sp_UDB014269_SH1211219.08FU_F_ITS2
Russula_velenovskyi_LC008526_SH1648042.08FU_F_ITS2
Russula_sp_UDB0757978_SH1648043.08FU_F_ITS2
Russula_sp_UDB016928_SH1519354.08FU_F_ITS2

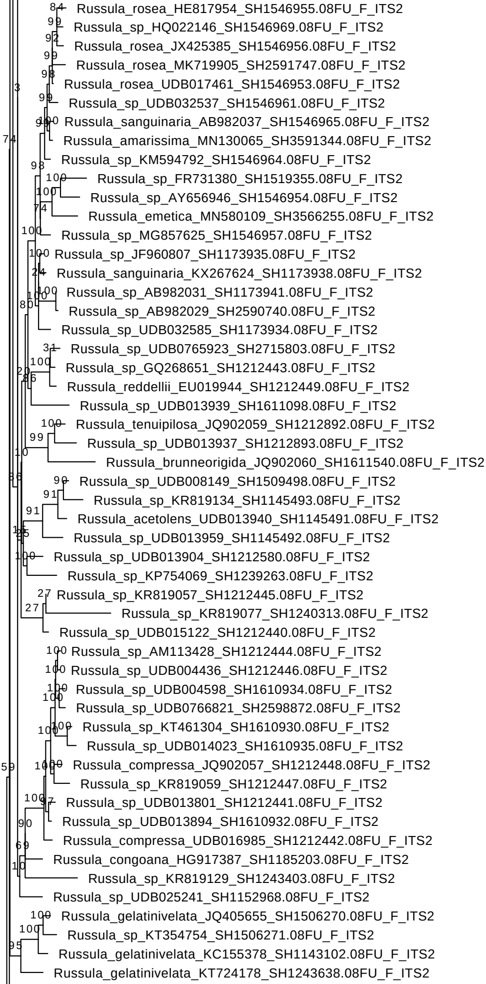
Russula_rosea_HE817954_SH1546955.08FU_F_ITS2
Russula_sp_HQ022146_SH1546969.08FU_F_ITS2
Russula_rosea_JX425385_SH1546956.08FU_F_ITS2
Russula_rosea_MK719905_SH2591747.08FU_F_ITS2
Russula_rosea_UDB017461_SH1546953.08FU_F_ITS2
Russula_sp_UDB032537_SH1546961.08FU_F_ITS2
Russula_sanguinaria_AB982037_SH1546965.08FU_F_ITS2
Russula_amarissima_MN130065_SH3591344.08FU_F_ITS2
Russula_sp_KM594792_SH1546964.08FU_F_ITS2
Russula_sp_FR731380_SH1519355.08FU_F_ITS2
Russula_sp_AY656946_SH1546954.08FU_F_ITS2
Russula_emetica_MN580109_SH3566255.08FU_F_ITS2
Russula_sp_MG857625_SH1546957.08FU_F_ITS2
Russula_sp_JF960807_SH1173935.08FU_F_ITS2
Russula_sanguinaria_KX267624_SH1173938.08FU_F_ITS2
Russula_sp_AB982031_SH1173941.08FU_F_ITS2
Russula_sp_AB982029_SH2590740.08FU_F_ITS2
Russula_sp_UDB032585_SH1173934.08FU_F_ITS2
Russula_sp_UDB0765923_SH2715803.08FU_F_ITS2
Russula_sp_GQ268651_SH1212443.08FU_F_ITS2
Russula_reddellii_EU019944_SH1212449.08FU_F_ITS2
Russula_sp_UDB013939_SH1611098.08FU_F_ITS2
Russula_tenuipilosa_JQ902059_SH1212892.08FU_F_ITS2
Russula_sp_UDB013937_SH1212893.08FU_F_ITS2
Russula_brunneorigida_JQ902060_SH1611540.08FU_F_ITS2
Russula_sp_UDB008149_SH1509498.08FU_F_ITS2
Russula_sp_KR819134_SH1145493.08FU_F_ITS2
Russula_acetolens_UDB013940_SH1145491.08FU_F_ITS2
Russula_sp_UDB013959_SH1145492.08FU_F_ITS2
Russula_sp_UDB013904_SH1212580.08FU_F_ITS2
Russula_sp_KP754069_SH1239263.08FU_F_ITS2
Russula_sp_KR819057_SH1212445.08FU_F_ITS2
Russula_sp_KR819077_SH1240313.08FU_F_ITS2
Russula_sp_UDB015122_SH1212440.08FU_F_ITS2
Russula_sp_AM113428_SH1212444.08FU_F_ITS2
Russula_sp_UDB004436_SH1212446.08FU_F_ITS2
Russula_sp_UDB004598_SH1610934.08FU_F_ITS2
Russula_sp_UDB0766821_SH2598872.08FU_F_ITS2
Russula_sp_KT461304_SH1610930.08FU_F_ITS2
Russula_sp_UDB014023_SH1610935.08FU_F_ITS2
Russula_compressa_JQ902057_SH1212448.08FU_F_ITS2
Russula_sp_KR819059_SH1212447.08FU_F_ITS2
Russula_sp_UDB013801_SH1212441.08FU_F_ITS2
Russula_sp_UDB013894_SH1610932.08FU_F_ITS2
Russula_compressa_UDB016985_SH1212442.08FU_F_ITS2
Russula_congoana_HG917387_SH1185203.08FU_F_ITS2
Russula_sp_KR819129_SH1243403.08FU_F_ITS2
Russula_sp_UDB025241_SH1152968.08FU_F_ITS2
Russula_gelatinivelata_JQ405655_SH1506270.08FU_F_ITS2
Russula_sp_KT354754_SH1506271.08FU_F_ITS2
Russula_gelatinivelata_KC155378_SH1143102.08FU_F_ITS2
Russula_gelatinivelata_KT724178_SH1243638.08FU_F_ITS2

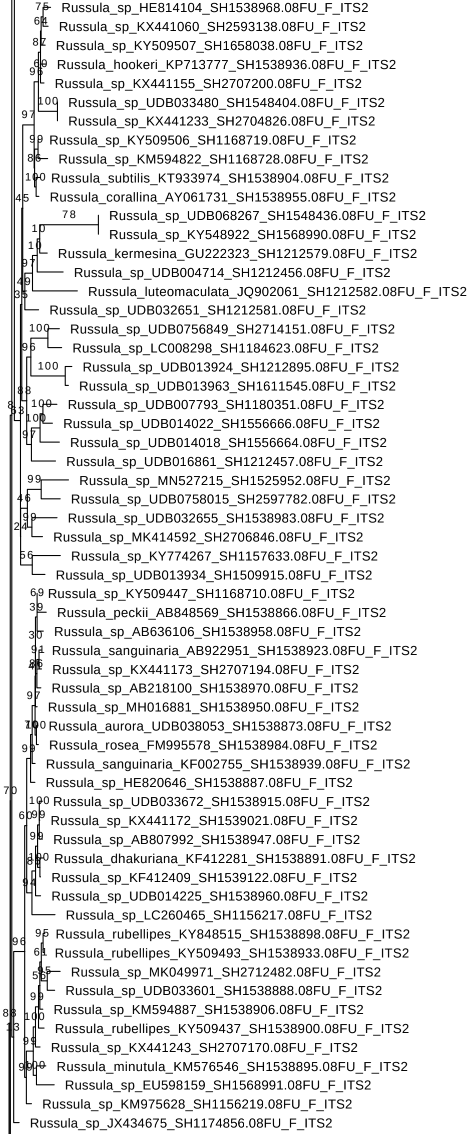
Russula_sp_HE814104_SH1538968.08FU_F_ITS2
Russula_sp_KX441060_SH2593138.08FU_F_ITS2
Russula_sp_KY509507_SH1658038.08FU_F_ITS2
Russula_hookeri_KP713777_SH1538936.08FU_F_ITS2
Russula_sp_KX441155_SH2707200.08FU_F_ITS2
Russula_sp_UDB033480_SH1548404.08FU_F_ITS2
Russula_sp_KX441233_SH2704826.08FU_F_ITS2
Russula_sp_KY509506_SH1168719.08FU_F_ITS2
Russula_sp_KM594822_SH1168728.08FU_F_ITS2
Russula_subtilis_KT933974_SH1538904.08FU_F_ITS2
Russula_corallina_AY061731_SH1538955.08FU_F_ITS2
Russula_sp_UDB068267_SH1548436.08FU_F_ITS2
Russula_sp_KY548922_SH1568990.08FU_F_ITS2
Russula_kermesina_GU222323_SH1212579.08FU_F_ITS2
Russula_sp_UDB004714_SH1212456.08FU_F_ITS2
Russula_luteomaculata_JQ902061_SH1212582.08FU_F_ITS2
Russula_sp_UDB032651_SH1212581.08FU_F_ITS2
Russula_sp_UDB0756849_SH2714151.08FU_F_ITS2
Russula_sp_LC008298_SH1184623.08FU_F_ITS2
Russula_sp_UDB013924_SH1212895.08FU_F_ITS2
Russula_sp_UDB013963_SH1611545.08FU_F_ITS2
Russula_sp_UDB007793_SH1180351.08FU_F_ITS2
Russula_sp_UDB014022_SH1556666.08FU_F_ITS2
Russula_sp_UDB014018_SH1556664.08FU_F_ITS2
Russula_sp_UDB016861_SH1212457.08FU_F_ITS2
Russula_sp_MN527215_SH1525952.08FU_F_ITS2
Russula_sp_UDB0758015_SH2597782.08FU_F_ITS2
Russula_sp_UDB032655_SH1538983.08FU_F_ITS2
Russula_sp_MK414592_SH2706846.08FU_F_ITS2
Russula_sp_KY774267_SH1157633.08FU_F_ITS2
Russula_sp_UDB013934_SH1509915.08FU_F_ITS2
Russula_sp_KY509447_SH1168710.08FU_F_ITS2
Russula_peckii_AB848569_SH1538866.08FU_F_ITS2
Russula_sp_AB636106_SH1538958.08FU_F_ITS2
Russula_sanguinaria_AB922951_SH1538923.08FU_F_ITS2
Russula_sp_KX441173_SH2707194.08FU_F_ITS2
Russula_sp_AB218100_SH1538970.08FU_F_ITS2
Russula_sp_MH016881_SH1538950.08FU_F_ITS2
Russula_aurora_UDB038053_SH1538873.08FU_F_ITS2
Russula_rosea_FM995578_SH1538984.08FU_F_ITS2
Russula_sanguinaria_KF002755_SH1538939.08FU_F_ITS2
Russula_sp_HE820646_SH1538887.08FU_F_ITS2
Russula_sp_UDB033672_SH1538915.08FU_F_ITS2
Russula_sp_KX441172_SH1539021.08FU_F_ITS2
Russula_sp_AB807992_SH1538947.08FU_F_ITS2
Russula_dhakuriana_KF412281_SH1538891.08FU_F_ITS2
Russula_sp_KF412409_SH1539122.08FU_F_ITS2
Russula_sp_UDB014225_SH1538960.08FU_F_ITS2
Russula_sp_LC260465_SH1156217.08FU_F_ITS2
Russula_rubellipes_KY848515_SH1538898.08FU_F_ITS2
Russula_rubellipes_KY509493_SH1538933.08FU_F_ITS2
Russula_sp_MK049971_SH2712482.08FU_F_ITS2
Russula_sp_UDB033601_SH1538888.08FU_F_ITS2
Russula_sp_KM594887_SH1538906.08FU_F_ITS2
Russula_rubellipes_KY509437_SH1538900.08FU_F_ITS2
Russula_sp_KX441243_SH2707170.08FU_F_ITS2
Russula_minutula_KM576546_SH1538895.08FU_F_ITS2
Russula_sp_EU598159_SH1568991.08FU_F_ITS2
Russula_sp_KM975628_SH1156219.08FU_F_ITS2
Russula_sp_JX434675_SH1174856.08FU_F_ITS2

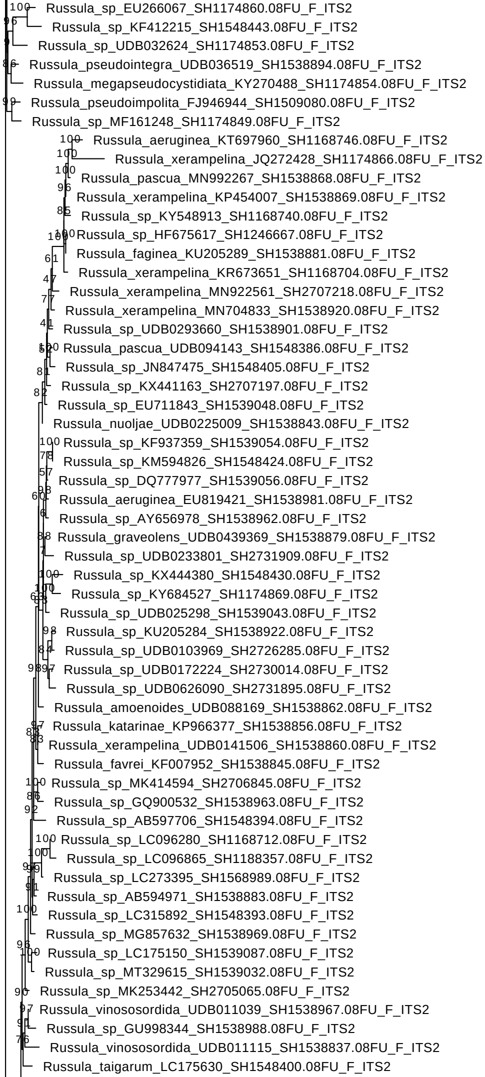
Russula_sp_EU266067_SH1174860.08FU_F_ITS2
Russula_sp_KF412215_SH1548443.08FU_F_ITS2
Russula_sp_UDB032624_SH1174853.08FU_F_ITS2
Russula_pseudointegra_UDB036519_SH1538894.08FU_F_ITS2
Russula_megapseudocystidiata_KY270488_SH1174854.08FU_F_ITS2
Russula_pseudoimpolita_FJ946944_SH1509080.08FU_F_ITS2
Russula_sp_MF161248_SH1174849.08FU_F_ITS2
Russula_aeruginea_KT697960_SH1168746.08FU_F_ITS2
Russula_xerampelina_JQ272428_SH1174866.08FU_F_ITS2
Russula_pascua_MN992267_SH1538868.08FU_F_ITS2
Russula_xerampelina_KP454007_SH1538869.08FU_F_ITS2
Russula_sp_KY548913_SH1168740.08FU_F_ITS2
Russula_sp_HF675617_SH1246667.08FU_F_ITS2
Russula_faginea_KU205289_SH1538881.08FU_F_ITS2
Russula_xerampelina_KR673651_SH1168704.08FU_F_ITS2
Russula_xerampelina_MN922561_SH2707218.08FU_F_ITS2
Russula_xerampelina_MN704833_SH1538920.08FU_F_ITS2
Russula_sp_UDB0293660_SH1538901.08FU_F_ITS2
Russula_pascua_UDB094143_SH1548386.08FU_F_ITS2
Russula_sp_JN847475_SH1548405.08FU_F_ITS2
Russula_sp_KX441163_SH2707197.08FU_F_ITS2
Russula_sp_EU711843_SH1539048.08FU_F_ITS2
Russula_nuoljae_UDB0225009_SH1538843.08FU_F_ITS2
Russula_sp_KF937359_SH1539054.08FU_F_ITS2
Russula_sp_KM594826_SH1548424.08FU_F_ITS2
Russula_sp_DQ777977_SH1539056.08FU_F_ITS2
Russula_aeruginea_EU819421_SH1538981.08FU_F_ITS2
Russula_sp_AY656978_SH1538962.08FU_F_ITS2
Russula_graveolens_UDB0439369_SH1538879.08FU_F_ITS2
Russula_sp_UDB0233801_SH2731909.08FU_F_ITS2
Russula_sp_KX444380_SH1548430.08FU_F_ITS2
Russula_sp_KY684527_SH1174869.08FU_F_ITS2
Russula_sp_UDB025298_SH1539043.08FU_F_ITS2
Russula_sp_KU205284_SH1538922.08FU_F_ITS2
Russula_sp_UDB0103969_SH2726285.08FU_F_ITS2
Russula_sp_UDB0172224_SH2730014.08FU_F_ITS2
Russula_sp_UDB0626090_SH2731895.08FU_F_ITS2
Russula_amoenoides_UDB088169_SH1538862.08FU_F_ITS2
Russula_katarinae_KP966377_SH1538856.08FU_F_ITS2
Russula_xerampelina_UDB0141506_SH1538860.08FU_F_ITS2
Russula_favrei_KF007952_SH1538845.08FU_F_ITS2
Russula_sp_MK414594_SH2706845.08FU_F_ITS2
Russula_sp_GQ900532_SH1538963.08FU_F_ITS2
Russula_sp_AB597706_SH1548394.08FU_F_ITS2
Russula_sp_LC096280_SH1168712.08FU_F_ITS2
Russula_sp_LC096865_SH1188357.08FU_F_ITS2
Russula_sp_LC273395_SH1568989.08FU_F_ITS2
Russula_sp_AB594971_SH1538883.08FU_F_ITS2
Russula_sp_LC315892_SH1548393.08FU_F_ITS2
Russula_sp_MG857632_SH1538969.08FU_F_ITS2
Russula_sp_LC175150_SH1539087.08FU_F_ITS2
Russula_sp_MT329615_SH1539032.08FU_F_ITS2
Russula_sp_MK253442_SH2705065.08FU_F_ITS2
Russula_vinososordida_UDB011039_SH1538967.08FU_F_ITS2
Russula_sp_GU998344_SH1538988.08FU_F_ITS2
Russula_vinososordida_UDB011115_SH1538837.08FU_F_ITS2
Russula_taigarum_LC175630_SH1548400.08FU_F_ITS2

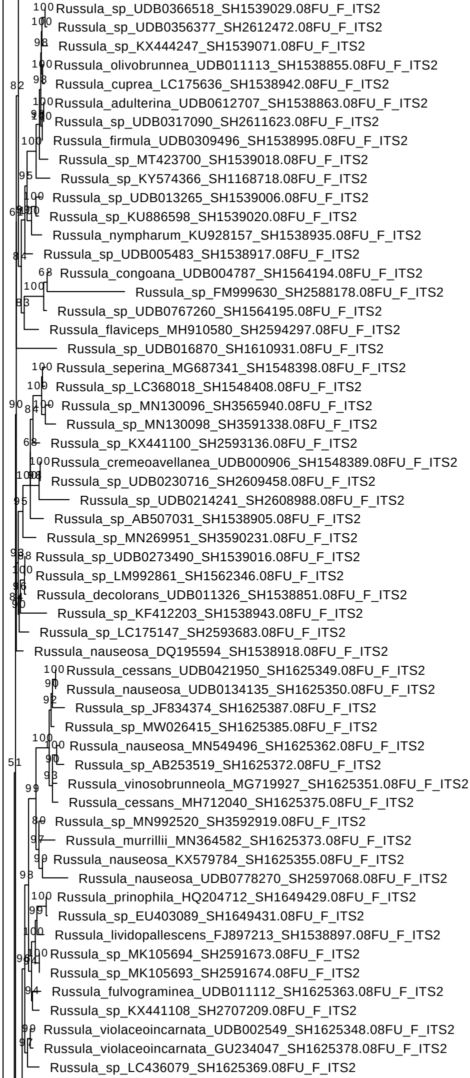
Russula_sp_UDB0366518_SH1539029.08FU_F_ITS2
Russula_sp_UDB0356377_SH2612472.08FU_F_ITS2
Russula_sp_KX444247_SH1539071.08FU_F_ITS2
Russula_olivobrunnea_UDB011113_SH1538855.08FU_F_ITS2
Russula_cuprea_LC175636_SH1538942.08FU_F_ITS2
Russula_adulterina_UDB0612707_SH1538863.08FU_F_ITS2
Russula_sp_UDB0317090_SH2611623.08FU_F_ITS2
Russula_firmula_UDB0309496_SH1538995.08FU_F_ITS2
Russula_sp_MT423700_SH1539018.08FU_F_ITS2
Russula_sp_KY574366_SH1168718.08FU_F_ITS2
Russula_sp_UDB013265_SH1539006.08FU_F_ITS2
Russula_sp_KU886598_SH1539020.08FU_F_ITS2
Russula_nympharum_KU928157_SH1538935.08FU_F_ITS2
Russula_sp_UDB005483_SH1538917.08FU_F_ITS2
Russula_congoana_UDB004787_SH1564194.08FU_F_ITS2
Russula_sp_FM999630_SH2588178.08FU_F_ITS2
Russula_sp_UDB0767260_SH1564195.08FU_F_ITS2
Russula_flaviceps_MH910580_SH2594297.08FU_F_ITS2
Russula_sp_UDB016870_SH1610931.08FU_F_ITS2
Russula_seperina_MG687341_SH1548398.08FU_F_ITS2
Russula_sp_LC368018_SH1548408.08FU_F_ITS2
Russula_sp_MN130096_SH3565940.08FU_F_ITS2
Russula_sp_MN130098_SH3591338.08FU_F_ITS2
Russula_sp_KX441100_SH2593136.08FU_F_ITS2
Russula_cremeoavellanea_UDB000906_SH1548389.08FU_F_ITS2
Russula_sp_UDB0230716_SH2609458.08FU_F_ITS2
Russula_sp_UDB0214241_SH2608988.08FU_F_ITS2
Russula_sp_AB507031_SH1538905.08FU_F_ITS2
Russula_sp_MN269951_SH3590231.08FU_F_ITS2
Russula_sp_UDB0273490_SH1539016.08FU_F_ITS2
Russula_sp_LM992861_SH1562346.08FU_F_ITS2
Russula_decolorans_UDB011326_SH1538851.08FU_F_ITS2
Russula_sp_KF412203_SH1538943.08FU_F_ITS2
Russula_sp_LC175147_SH2593683.08FU_F_ITS2
Russula_nauseosa_DQ195594_SH1538918.08FU_F_ITS2
Russula_cessans_UDB0421950_SH1625349.08FU_F_ITS2
Russula_nauseosa_UDB0134135_SH1625350.08FU_F_ITS2
Russula_sp_JF834374_SH1625387.08FU_F_ITS2
Russula_sp_MW026415_SH1625385.08FU_F_ITS2
Russula_nauseosa_MN549496_SH1625362.08FU_F_ITS2
Russula_sp_AB253519_SH1625372.08FU_F_ITS2
Russula_vinosobrunneola_MG719927_SH1625351.08FU_F_ITS2
Russula_cessans_MH712040_SH1625375.08FU_F_ITS2
Russula_sp_MN992520_SH3592919.08FU_F_ITS2
Russula_murrillii_MN364582_SH1625373.08FU_F_ITS2
Russula_nauseosa_KX579784_SH1625355.08FU_F_ITS2
Russula_nauseosa_UDB0778270_SH2597068.08FU_F_ITS2
Russula_prinophila_HQ204712_SH1649429.08FU_F_ITS2
Russula_sp_EU403089_SH1649431.08FU_F_ITS2
Russula_lividopallescens_FJ897213_SH1538897.08FU_F_ITS2
Russula_sp_MK105694_SH2591673.08FU_F_ITS2
Russula_sp_MK105693_SH2591674.08FU_F_ITS2
Russula_fulvograminea_UDB011112_SH1625363.08FU_F_ITS2
Russula_sp_KX441108_SH2707209.08FU_F_ITS2
Russula_violaceoincarnata_UDB002549_SH1625348.08FU_F_ITS2
Russula_violaceoincarnata_GU234047_SH1625378.08FU_F_ITS2
Russula_sp_LC436079_SH1625369.08FU_F_ITS2

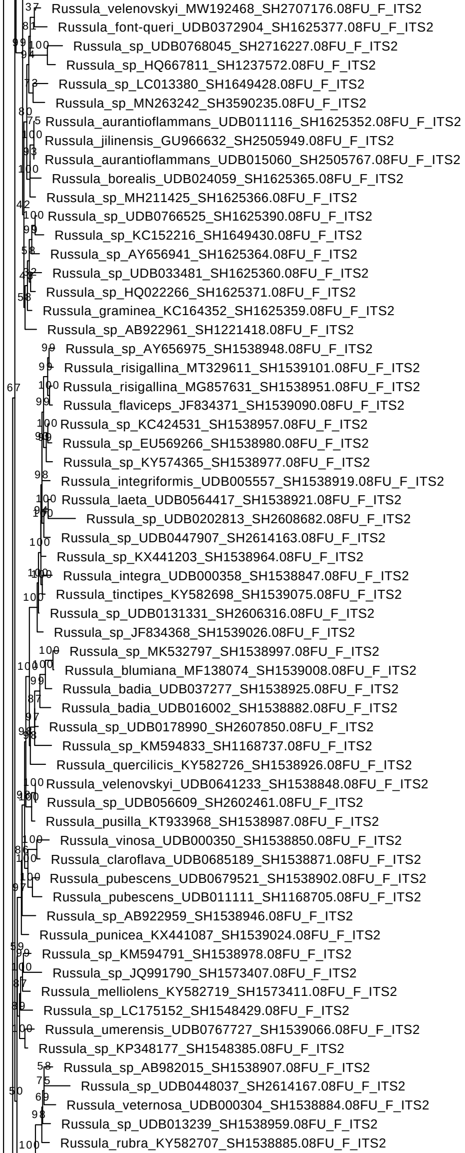
Russula_velenovskyi_MW192468_SH2707176.08FU_F_ITS2
Russula_font-queri_UDB0372904_SH1625377.08FU_F_ITS2
Russula_sp_UDB0768045_SH2716227.08FU_F_ITS2
Russula_sp_HQ667811_SH1237572.08FU_F_ITS2
Russula_sp_LC013380_SH1649428.08FU_F_ITS2
Russula_sp_MN263242_SH3590235.08FU_F_ITS2
Russula_aurantioflammans_UDB011116_SH1625352.08FU_F_ITS2
Russula_jilinensis_GU966632_SH2505949.08FU_F_ITS2
Russula_aurantioflammans_UDB015060_SH2505767.08FU_F_ITS2
Russula_borealis_UDB024059_SH1625365.08FU_F_ITS2
Russula_sp_MH211425_SH1625366.08FU_F_ITS2
Russula_sp_UDB0766525_SH1625390.08FU_F_ITS2
Russula_sp_KC152216_SH1649430.08FU_F_ITS2
Russula_sp_AY656941_SH1625364.08FU_F_ITS2
Russula_sp_UDB033481_SH1625360.08FU_F_ITS2
Russula_sp_HQ022266_SH1625371.08FU_F_ITS2
Russula_graminea_KC164352_SH1625359.08FU_F_ITS2
Russula_sp_AB922961_SH1221418.08FU_F_ITS2
Russula_sp_AY656975_SH1538948.08FU_F_ITS2
Russula_risigallina_MT329611_SH1539101.08FU_F_ITS2
Russula_risigallina_MG857631_SH1538951.08FU_F_ITS2
Russula_flaviceps_JF834371_SH1539090.08FU_F_ITS2
Russula_sp_KC424531_SH1538957.08FU_F_ITS2
Russula_sp_EU569266_SH1538980.08FU_F_ITS2
Russula_sp_KY574365_SH1538977.08FU_F_ITS2
Russula_integriformis_UDB005557_SH1538919.08FU_F_ITS2
Russula_laeta_UDB0564417_SH1538921.08FU_F_ITS2
Russula_sp_UDB0202813_SH2608682.08FU_F_ITS2
Russula_sp_UDB0447907_SH2614163.08FU_F_ITS2
Russula_sp_KX441203_SH1538964.08FU_F_ITS2
Russula_integra_UDB000358_SH1538847.08FU_F_ITS2
Russula_tinctipes_KY582698_SH1539075.08FU_F_ITS2
Russula_sp_UDB0131331_SH2606316.08FU_F_ITS2
Russula_sp_JF834368_SH1539026.08FU_F_ITS2
Russula_sp_MK532797_SH1538997.08FU_F_ITS2
Russula_blumiana_MF138074_SH1539008.08FU_F_ITS2
Russula_badia_UDB037277_SH1538925.08FU_F_ITS2
Russula_badia_UDB016002_SH1538882.08FU_F_ITS2
Russula_sp_UDB0178990_SH2607850.08FU_F_ITS2
Russula_sp_KM594833_SH1168737.08FU_F_ITS2
Russula_quercilicis_KY582726_SH1538926.08FU_F_ITS2
Russula_velenovskyi_UDB0641233_SH1538848.08FU_F_ITS2
Russula_sp_UDB056609_SH2602461.08FU_F_ITS2
Russula_pusilla_KT933968_SH1538987.08FU_F_ITS2
Russula_vinosa_UDB000350_SH1538850.08FU_F_ITS2
Russula_claroflava_UDB0685189_SH1538871.08FU_F_ITS2
Russula_pubescens_UDB0679521_SH1538902.08FU_F_ITS2
Russula_pubescens_UDB011111_SH1168705.08FU_F_ITS2
Russula_sp_AB922959_SH1538946.08FU_F_ITS2
Russula_punicea_KX441087_SH1539024.08FU_F_ITS2
Russula_sp_KM594791_SH1538978.08FU_F_ITS2
Russula_sp_JQ991790_SH1573407.08FU_F_ITS2
Russula_melliolens_KY582719_SH1573411.08FU_F_ITS2
Russula_sp_LC175152_SH1548429.08FU_F_ITS2
Russula_umerensis_UDB0767727_SH1539066.08FU_F_ITS2
Russula_sp_KP348177_SH1548385.08FU_F_ITS2
Russula_sp_AB982015_SH1538907.08FU_F_ITS2
Russula_sp_UDB0448037_SH2614167.08FU_F_ITS2
Russula_veternosa_UDB000304_SH1538884.08FU_F_ITS2
Russula_sp_UDB013239_SH1538959.08FU_F_ITS2
Russula_rubra_KY582707_SH1538885.08FU_F_ITS2

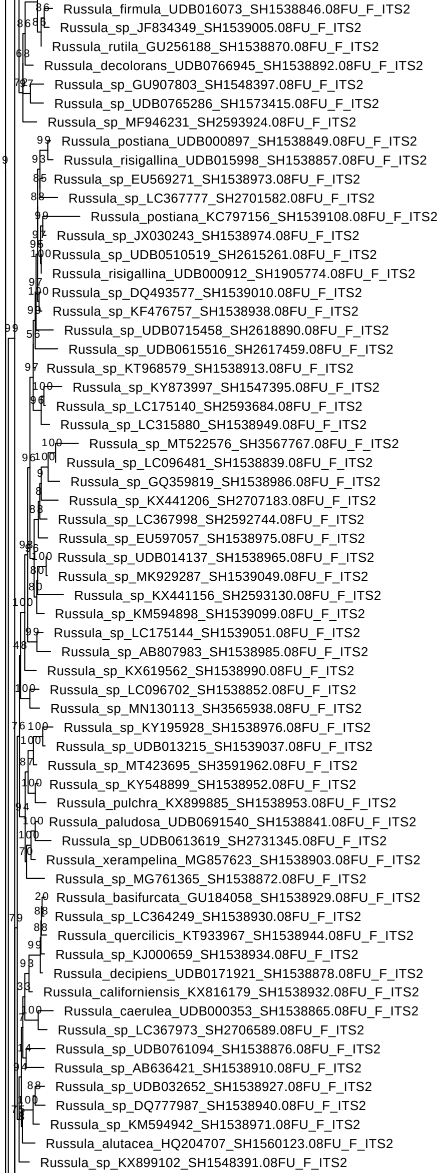
Russula_firmula_UDB016073_SH1538846.08FU_F_ITS2
Russula_sp_JF834349_SH1539005.08FU_F_ITS2
Russula_rutila_GU256188_SH1538870.08FU_F_ITS2
Russula_decolorans_UDB0766945_SH1538892.08FU_F_ITS2
Russula_sp_GU907803_SH1548397.08FU_F_ITS2
Russula_sp_UDB0765286_SH1573415.08FU_F_ITS2
Russula_sp_MF946231_SH2593924.08FU_F_ITS2
Russula_postiana_UDB000897_SH1538849.08FU_F_ITS2
Russula_risigallina_UDB015998_SH1538857.08FU_F_ITS2
Russula_sp_EU569271_SH1538973.08FU_F_ITS2
Russula_sp_LC367777_SH2701582.08FU_F_ITS2
Russula_postiana_KC797156_SH1539108.08FU_F_ITS2
Russula_sp_JX030243_SH1538974.08FU_F_ITS2
Russula_sp_UDB0510519_SH2615261.08FU_F_ITS2
Russula_risigallina_UDB000912_SH1905774.08FU_F_ITS2
Russula_sp_DQ493577_SH1539010.08FU_F_ITS2
Russula_sp_KF476757_SH1538938.08FU_F_ITS2
Russula_sp_UDB0715458_SH2618890.08FU_F_ITS2
Russula_sp_UDB0615516_SH2617459.08FU_F_ITS2
Russula_sp_KT968579_SH1538913.08FU_F_ITS2
Russula_sp_KY873997_SH1547395.08FU_F_ITS2
Russula_sp_LC175140_SH2593684.08FU_F_ITS2
Russula_sp_LC315880_SH1538949.08FU_F_ITS2
Russula_sp_MT522576_SH3567767.08FU_F_ITS2
Russula_sp_LC096481_SH1538839.08FU_F_ITS2
Russula_sp_GQ359819_SH1538986.08FU_F_ITS2
Russula_sp_KX441206_SH2707183.08FU_F_ITS2
Russula_sp_LC367998_SH2592744.08FU_F_ITS2
Russula_sp_EU597057_SH1538975.08FU_F_ITS2
Russula_sp_UDB014137_SH1538965.08FU_F_ITS2
Russula_sp_MK929287_SH1539049.08FU_F_ITS2
Russula_sp_KX441156_SH2593130.08FU_F_ITS2
Russula_sp_KM594898_SH1539099.08FU_F_ITS2
Russula_sp_LC175144_SH1539051.08FU_F_ITS2
Russula_sp_AB807983_SH1538985.08FU_F_ITS2
Russula_sp_KX619562_SH1538990.08FU_F_ITS2
Russula_sp_LC096702_SH1538852.08FU_F_ITS2
Russula_sp_MN130113_SH3565938.08FU_F_ITS2
Russula_sp_KY195928_SH1538976.08FU_F_ITS2
Russula_sp_UDB013215_SH1539037.08FU_F_ITS2
Russula_sp_MT423695_SH3591962.08FU_F_ITS2
Russula_sp_KY548899_SH1538952.08FU_F_ITS2
Russula_pulchra_KX899885_SH1538953.08FU_F_ITS2
Russula_paludosa_UDB0691540_SH1538841.08FU_F_ITS2
Russula_sp_UDB0613619_SH2731345.08FU_F_ITS2
Russula_xerampelina_MG857623_SH1538903.08FU_F_ITS2
Russula_sp_MG761365_SH1538872.08FU_F_ITS2
Russula_basifurcata_GU184058_SH1538929.08FU_F_ITS2
Russula_sp_LC364249_SH1538930.08FU_F_ITS2
Russula_quercilicis_KT933967_SH1538944.08FU_F_ITS2
Russula_sp_KJ000659_SH1538934.08FU_F_ITS2
Russula_decipiens_UDB0171921_SH1538878.08FU_F_ITS2
Russula_californiensis_KX816179_SH1538932.08FU_F_ITS2
Russula_caerulea_UDB000353_SH1538865.08FU_F_ITS2
Russula_sp_LC367973_SH2706589.08FU_F_ITS2
Russula_sp_UDB0761094_SH1538876.08FU_F_ITS2
Russula_sp_AB636421_SH1538910.08FU_F_ITS2
Russula_sp_UDB032652_SH1538927.08FU_F_ITS2
Russula_sp_DQ777987_SH1538940.08FU_F_ITS2
Russula_sp_KM594942_SH1538971.08FU_F_ITS2
Russula_alutacea_HQ204707_SH1560123.08FU_F_ITS2
Russula_sp_KX899102_SH1548391.08FU_F_ITS2

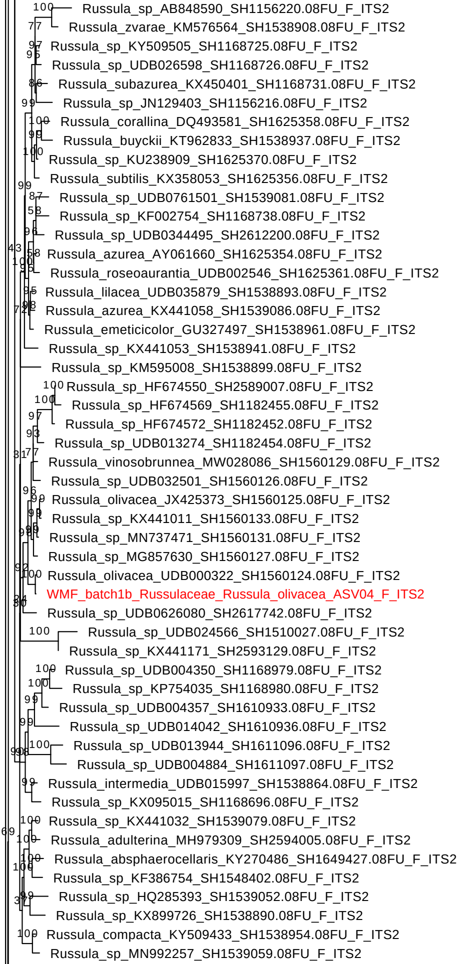
Russula_sp_AB848590_SH1156220.08FU_F_ITS2
Russula_zvarae_KM576564_SH1538908.08FU_F_ITS2
Russula_sp_KY509505_SH1168725.08FU_F_ITS2
Russula_sp_UDB026598_SH1168726.08FU_F_ITS2
Russula_subazurea_KX450401_SH1168731.08FU_F_ITS2
Russula_sp_JN129403_SH1156216.08FU_F_ITS2
Russula_corallina_DQ493581_SH1625358.08FU_F_ITS2
Russula_buyckii_KT962833_SH1538937.08FU_F_ITS2
Russula_sp_KU238909_SH1625370.08FU_F_ITS2
Russula_subtilis_KX358053_SH1625356.08FU_F_ITS2
Russula_sp_UDB0761501_SH1539081.08FU_F_ITS2
Russula_sp_KF002754_SH1168738.08FU_F_ITS2
Russula_sp_UDB0344495_SH2612200.08FU_F_ITS2
Russula_azurea_AY061660_SH1625354.08FU_F_ITS2
Russula_roseoaurantia_UDB002546_SH1625361.08FU_F_ITS2
Russula_lilacea_UDB035879_SH1538893.08FU_F_ITS2
Russula_azurea_KX441058_SH1539086.08FU_F_ITS2
Russula_emeticicolor_GU327497_SH1538961.08FU_F_ITS2
Russula_sp_KX441053_SH1538941.08FU_F_ITS2
Russula_sp_KM595008_SH1538899.08FU_F_ITS2
Russula_sp_HF674550_SH2589007.08FU_F_ITS2
Russula_sp_HF674569_SH1182455.08FU_F_ITS2
Russula_sp_HF674572_SH1182452.08FU_F_ITS2
Russula_sp_UDB013274_SH1182454.08FU_F_ITS2
Russula_vinosobrunnea_MW028086_SH1560129.08FU_F_ITS2
Russula_sp_UDB032501_SH1560126.08FU_F_ITS2
Russula_olivacea_JX425373_SH1560125.08FU_F_ITS2
Russula_sp_KX441011_SH1560133.08FU_F_ITS2
Russula_sp_MN737471_SH1560131.08FU_F_ITS2
Russula_sp_MG857630_SH1560127.08FU_F_ITS2
Russula_olivacea_UDB000322_SH1560124.08FU_F_ITS2
WMF_batch1b_Russulaceae_Russula_olivacea_ASV04_F_ITS2
Russula_sp_UDB0626080_SH2617742.08FU_F_ITS2
Russula_sp_UDB024566_SH1510027.08FU_F_ITS2
Russula_sp_KX441171_SH2593129.08FU_F_ITS2
Russula_sp_UDB004350_SH1168979.08FU_F_ITS2
Russula_sp_KP754035_SH1168980.08FU_F_ITS2
Russula_sp_UDB004357_SH1610933.08FU_F_ITS2
Russula_sp_UDB014042_SH1610936.08FU_F_ITS2
Russula_sp_UDB013944_SH1611096.08FU_F_ITS2
Russula_sp_UDB004884_SH1611097.08FU_F_ITS2
Russula_intermedia_UDB015997_SH1538864.08FU_F_ITS2
Russula_sp_KX095015_SH1168696.08FU_F_ITS2
Russula_sp_KX441032_SH1539079.08FU_F_ITS2
Russula_adulterina_MH979309_SH2594005.08FU_F_ITS2
Russula_absphaerocellaris_KY270486_SH1649427.08FU_F_ITS2
Russula_sp_KF386754_SH1548402.08FU_F_ITS2
Russula_sp_HQ285393_SH1539052.08FU_F_ITS2
Russula_sp_KX899726_SH1538890.08FU_F_ITS2
Russula_compacta_KY509433_SH1538954.08FU_F_ITS2
Russula_sp_MN992257_SH1539059.08FU_F_ITS2

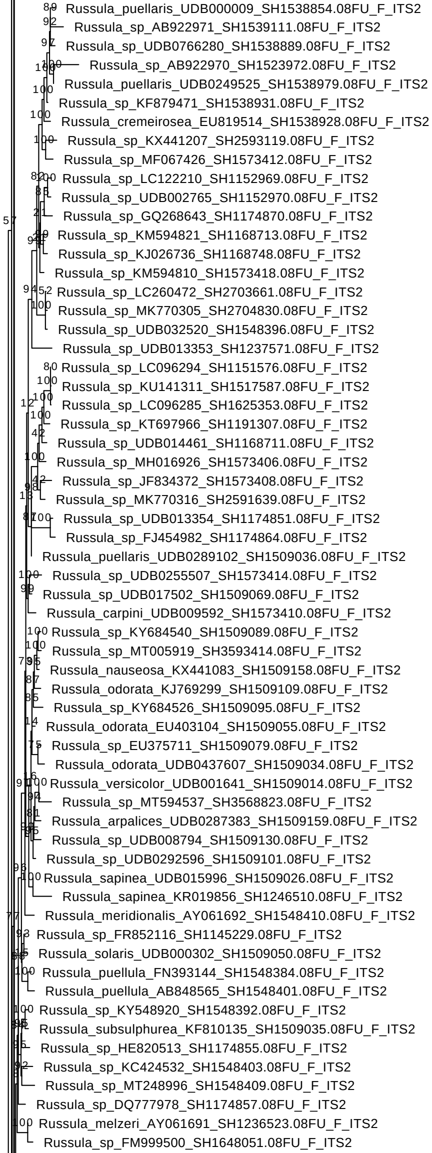
Russula_puellaris_UDB000009_SH1538854.08FU_F_ITS2
Russula_sp_AB922971_SH1539111.08FU_F_ITS2
Russula_sp_UDB0766280_SH1538889.08FU_F_ITS2
Russula_sp_AB922970_SH1523972.08FU_F_ITS2
Russula_puellaris_UDB0249525_SH1538979.08FU_F_ITS2
Russula_sp_KF879471_SH1538931.08FU_F_ITS2
Russula_cremeirosea_EU819514_SH1538928.08FU_F_ITS2
Russula_sp_KX441207_SH2593119.08FU_F_ITS2
Russula_sp_MF067426_SH1573412.08FU_F_ITS2
Russula_sp_LC122210_SH1152969.08FU_F_ITS2
Russula_sp_UDB002765_SH1152970.08FU_F_ITS2
Russula_sp_GQ268643_SH1174870.08FU_F_ITS2
Russula_sp_KM594821_SH1168713.08FU_F_ITS2
Russula_sp_KJ026736_SH1168748.08FU_F_ITS2
Russula_sp_KM594810_SH1573418.08FU_F_ITS2
Russula_sp_LC260472_SH2703661.08FU_F_ITS2
Russula_sp_MK770305_SH2704830.08FU_F_ITS2
Russula_sp_UDB032520_SH1548396.08FU_F_ITS2
Russula_sp_UDB013353_SH1237571.08FU_F_ITS2
Russula_sp_LC096294_SH1151576.08FU_F_ITS2
Russula_sp_KU141311_SH1517587.08FU_F_ITS2
Russula_sp_LC096285_SH1625353.08FU_F_ITS2
Russula_sp_KT697966_SH1191307.08FU_F_ITS2
Russula_sp_UDB014461_SH1168711.08FU_F_ITS2
Russula_sp_MH016926_SH1573406.08FU_F_ITS2
Russula_sp_JF834372_SH1573408.08FU_F_ITS2
Russula_sp_MK770316_SH2591639.08FU_F_ITS2
Russula_sp_UDB013354_SH1174851.08FU_F_ITS2
Russula_sp_FJ454982_SH1174864.08FU_F_ITS2
Russula_puellaris_UDB0289102_SH1509036.08FU_F_ITS2
Russula_sp_UDB0255507_SH1573414.08FU_F_ITS2
Russula_sp_UDB017502_SH1509069.08FU_F_ITS2
Russula_carpini_UDB009592_SH1573410.08FU_F_ITS2
Russula_sp_KY684540_SH1509089.08FU_F_ITS2
Russula_sp_MT005919_SH3593414.08FU_F_ITS2
Russula_nauseosa_KX441083_SH1509158.08FU_F_ITS2
Russula_odorata_KJ769299_SH1509109.08FU_F_ITS2
Russula_sp_KY684526_SH1509095.08FU_F_ITS2
Russula_odorata_EU403104_SH1509055.08FU_F_ITS2
Russula_sp_EU375711_SH1509079.08FU_F_ITS2
Russula_odorata_UDB0437607_SH1509034.08FU_F_ITS2
Russula_versicolor_UDB001641_SH1509014.08FU_F_ITS2
Russula_sp_MT594537_SH3568823.08FU_F_ITS2
Russula_arpalices_UDB0287383_SH1509159.08FU_F_ITS2
Russula_sp_UDB008794_SH1509130.08FU_F_ITS2
Russula_sp_UDB0292596_SH1509101.08FU_F_ITS2
Russula_sapinea_UDB015996_SH1509026.08FU_F_ITS2
Russula_sapinea_KR019856_SH1246510.08FU_F_ITS2
Russula_meridionalis_AY061692_SH1548410.08FU_F_ITS2
Russula_sp_FR852116_SH1145229.08FU_F_ITS2
Russula_solaris_UDB000302_SH1509050.08FU_F_ITS2
Russula_puellula_FN393144_SH1548384.08FU_F_ITS2
Russula_puellula_AB848565_SH1548401.08FU_F_ITS2
Russula_sp_KY548920_SH1548392.08FU_F_ITS2
Russula_subsulphurea_KF810135_SH1509035.08FU_F_ITS2
Russula_sp_HE820513_SH1174855.08FU_F_ITS2
Russula_sp_KC424532_SH1548403.08FU_F_ITS2
Russula_sp_MT248996_SH1548409.08FU_F_ITS2
Russula_sp_DQ777978_SH1174857.08FU_F_ITS2
Russula_melzeri_AY061691_SH1236523.08FU_F_ITS2
Russula_sp_FM999500_SH1648051.08FU_F_ITS2

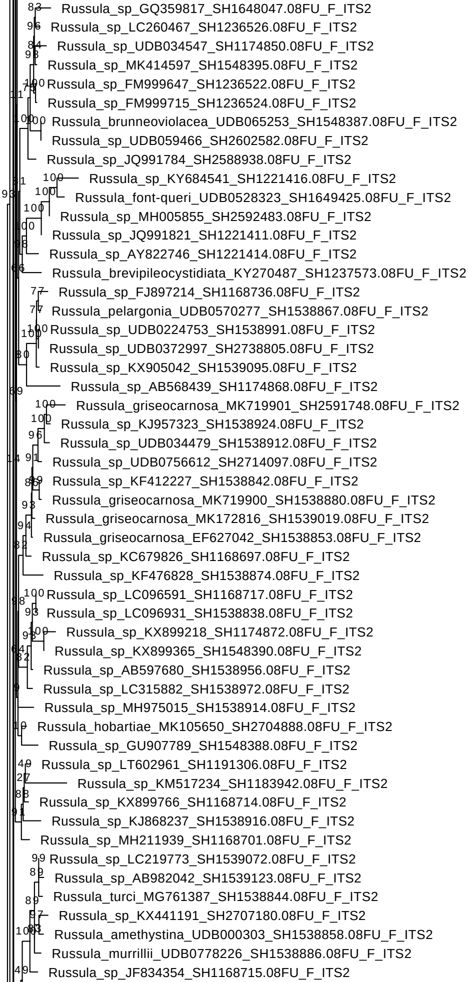
Russula_sp_GQ359817_SH1648047.08FU_F_ITS2
Russula_sp_LC260467_SH1236526.08FU_F_ITS2
Russula_sp_UDB034547_SH1174850.08FU_F_ITS2
Russula_sp_MK414597_SH1548395.08FU_F_ITS2
Russula_sp_FM999647_SH1236522.08FU_F_ITS2
Russula_sp_FM999715_SH1236524.08FU_F_ITS2
Russula_brunneoviolacea_UDB065253_SH1548387.08FU_F_ITS2
Russula_sp_UDB059466_SH2602582.08FU_F_ITS2
Russula_sp_JQ991784_SH2588938.08FU_F_ITS2
Russula_sp_KY684541_SH1221416.08FU_F_ITS2
Russula_font-queri_UDB0528323_SH1649425.08FU_F_ITS2
Russula_sp_MH005855_SH2592483.08FU_F_ITS2
Russula_sp_JQ991821_SH1221411.08FU_F_ITS2
Russula_sp_AY822746_SH1221414.08FU_F_ITS2
Russula_brevipileocystidiata_KY270487_SH1237573.08FU_F_ITS2
Russula_sp_FJ897214_SH1168736.08FU_F_ITS2
Russula_pelargonia_UDB0570277_SH1538867.08FU_F_ITS2
Russula_sp_UDB0224753_SH1538991.08FU_F_ITS2
Russula_sp_UDB0372997_SH2738805.08FU_F_ITS2
Russula_sp_KX905042_SH1539095.08FU_F_ITS2
Russula_sp_AB568439_SH1174868.08FU_F_ITS2
Russula_griseocarnosa_MK719901_SH2591748.08FU_F_ITS2
Russula_sp_KJ957323_SH1538924.08FU_F_ITS2
Russula_sp_UDB034479_SH1538912.08FU_F_ITS2
Russula_sp_UDB0756612_SH2714097.08FU_F_ITS2
Russula_sp_KF412227_SH1538842.08FU_F_ITS2
Russula_griseocarnosa_MK719900_SH1538880.08FU_F_ITS2
Russula_griseocarnosa_MK172816_SH1539019.08FU_F_ITS2
Russula_griseocarnosa_EF627042_SH1538853.08FU_F_ITS2
Russula_sp_KC679826_SH1168697.08FU_F_ITS2
Russula_sp_KF476828_SH1538874.08FU_F_ITS2
Russula_sp_LC096591_SH1168717.08FU_F_ITS2
Russula_sp_LC096931_SH1538838.08FU_F_ITS2
Russula_sp_KX899218_SH1174872.08FU_F_ITS2
Russula_sp_KX899365_SH1548390.08FU_F_ITS2
Russula_sp_AB597680_SH1538956.08FU_F_ITS2
Russula_sp_LC315882_SH1538972.08FU_F_ITS2
Russula_sp_MH975015_SH1538914.08FU_F_ITS2
Russula_hobartiae_MK105650_SH2704888.08FU_F_ITS2
Russula_sp_GU907789_SH1548388.08FU_F_ITS2
Russula_sp_LT602961_SH1191306.08FU_F_ITS2
Russula_sp_KM517234_SH1183942.08FU_F_ITS2
Russula_sp_KX899766_SH1168714.08FU_F_ITS2
Russula_sp_KJ868237_SH1538916.08FU_F_ITS2
Russula_sp_MH211939_SH1168701.08FU_F_ITS2
Russula_sp_LC219773_SH1539072.08FU_F_ITS2
Russula_sp_AB982042_SH1539123.08FU_F_ITS2
Russula_turci_MG761387_SH1538844.08FU_F_ITS2
Russula_sp_KX441191_SH2707180.08FU_F_ITS2
Russula_amethystina_UDB000303_SH1538858.08FU_F_ITS2
Russula_murrillii_UDB0778226_SH1538886.08FU_F_ITS2
Russula_sp_JF834354_SH1168715.08FU_F_ITS2

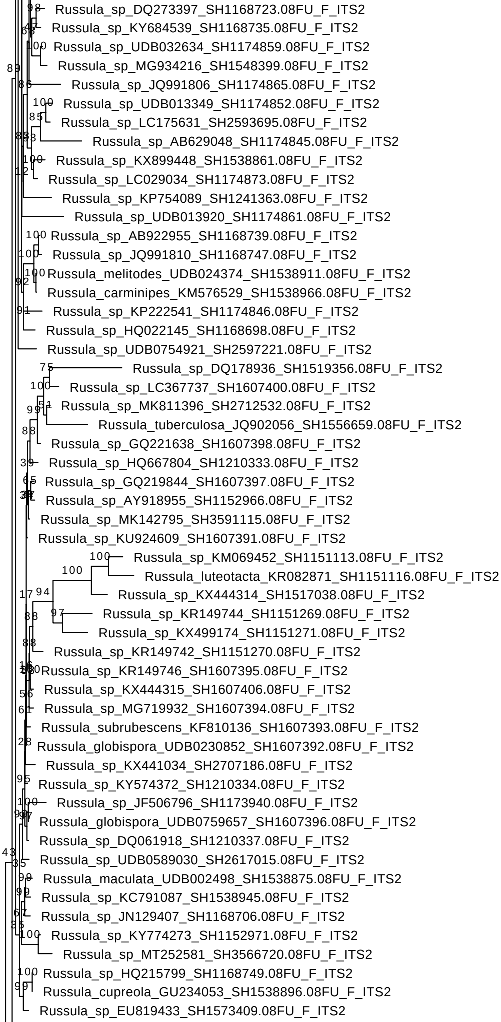
Russula_sp_DQ273397_SH1168723.08FU_F_ITS2
Russula_sp_KY684539_SH1168735.08FU_F_ITS2
Russula_sp_UDB032634_SH1174859.08FU_F_ITS2
Russula_sp_MG934216_SH1548399.08FU_F_ITS2
Russula_sp_JQ991806_SH1174865.08FU_F_ITS2
Russula_sp_UDB013349_SH1174852.08FU_F_ITS2
Russula_sp_LC175631_SH2593695.08FU_F_ITS2
Russula_sp_AB629048_SH1174845.08FU_F_ITS2
Russula_sp_KX899448_SH1538861.08FU_F_ITS2
Russula_sp_LC029034_SH1174873.08FU_F_ITS2
Russula_sp_KP754089_SH1241363.08FU_F_ITS2
Russula_sp_UDB013920_SH1174861.08FU_F_ITS2
Russula_sp_AB922955_SH1168739.08FU_F_ITS2
Russula_sp_JQ991810_SH1168747.08FU_F_ITS2
Russula_melitodes_UDB024374_SH1538911.08FU_F_ITS2
Russula_carminipes_KM576529_SH1538966.08FU_F_ITS2
Russula_sp_KP222541_SH1174846.08FU_F_ITS2
Russula_sp_HQ022145_SH1168698.08FU_F_ITS2
Russula_sp_UDB0754921_SH2597221.08FU_F_ITS2
Russula_sp_DQ178936_SH1519356.08FU_F_ITS2
Russula_sp_LC367737_SH1607400.08FU_F_ITS2
Russula_sp_MK811396_SH2712532.08FU_F_ITS2
Russula_tuberculosa_JQ902056_SH1556659.08FU_F_ITS2
Russula_sp_GQ221638_SH1607398.08FU_F_ITS2
Russula_sp_HQ667804_SH1210333.08FU_F_ITS2
Russula_sp_GQ219844_SH1607397.08FU_F_ITS2
Russula_sp_AY918955_SH1152966.08FU_F_ITS2
Russula_sp_MK142795_SH3591115.08FU_F_ITS2
Russula_sp_KU924609_SH1607391.08FU_F_ITS2
Russula_sp_KM069452_SH1151113.08FU_F_ITS2
Russula_luteotacta_KR082871_SH1151116.08FU_F_ITS2
Russula_sp_KX444314_SH1517038.08FU_F_ITS2
Russula_sp_KR149744_SH1151269.08FU_F_ITS2
Russula_sp_KX499174_SH1151271.08FU_F_ITS2
Russula_sp_KR149742_SH1151270.08FU_F_ITS2
Russula_sp_KR149746_SH1607395.08FU_F_ITS2
Russula_sp_KX444315_SH1607406.08FU_F_ITS2
Russula_sp_MG719932_SH1607394.08FU_F_ITS2
Russula_subrubescens_KF810136_SH1607393.08FU_F_ITS2
Russula_globispora_UDB0230852_SH1607392.08FU_F_ITS2
Russula_sp_KX441034_SH2707186.08FU_F_ITS2
Russula_sp_KY574372_SH1210334.08FU_F_ITS2
Russula_sp_JF506796_SH1173940.08FU_F_ITS2
Russula_globispora_UDB0759657_SH1607396.08FU_F_ITS2
Russula_sp_DQ061918_SH1210337.08FU_F_ITS2
Russula_sp_UDB0589030_SH2617015.08FU_F_ITS2
Russula_maculata_UDB002498_SH1538875.08FU_F_ITS2
Russula_sp_KC791087_SH1538945.08FU_F_ITS2
Russula_sp_JN129407_SH1168706.08FU_F_ITS2
Russula_sp_KY774273_SH1152971.08FU_F_ITS2
Russula_sp_MT252581_SH3566720.08FU_F_ITS2
Russula_sp_HQ215799_SH1168749.08FU_F_ITS2
Russula_cupreola_GU234053_SH1538896.08FU_F_ITS2
Russula_sp_EU819433_SH1573409.08FU_F_ITS2

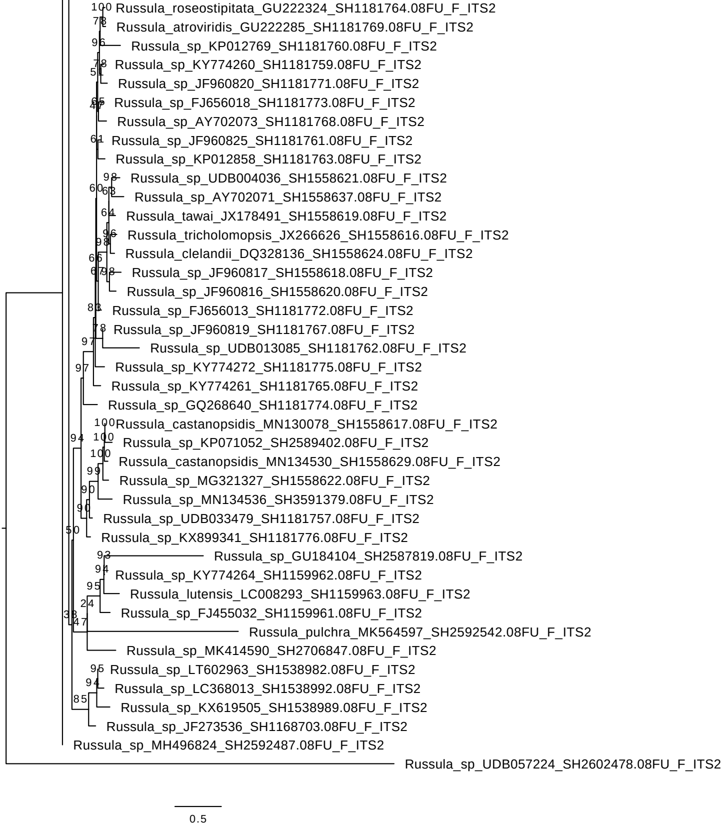
Russula_roseostipitata_GU222324_SH1181764.08FU_F_ITS2
Russula_atroviridis_GU222285_SH1181769.08FU_F_ITS2
Russula_sp_KP012769_SH1181760.08FU_F_ITS2
Russula_sp_KY774260_SH1181759.08FU_F_ITS2
Russula_sp_JF960820_SH1181771.08FU_F_ITS2
Russula_sp_FJ656018_SH1181773.08FU_F_ITS2
Russula_sp_AY702073_SH1181768.08FU_F_ITS2
Russula_sp_JF960825_SH1181761.08FU_F_ITS2
Russula_sp_KP012858_SH1181763.08FU_F_ITS2
Russula_sp_UDB004036_SH1558621.08FU_F_ITS2
Russula_sp_AY702071_SH1558637.08FU_F_ITS2
Russula_tawai_JX178491_SH1558619.08FU_F_ITS2
Russula_tricholomopsis_JX266626_SH1558616.08FU_F_ITS2
Russula_clelandii_DQ328136_SH1558624.08FU_F_ITS2
Russula_sp_JF960817_SH1558618.08FU_F_ITS2
Russula_sp_JF960816_SH1558620.08FU_F_ITS2
Russula_sp_FJ656013_SH1181772.08FU_F_ITS2
Russula_sp_JF960819_SH1181767.08FU_F_ITS2
Russula_sp_UDB013085_SH1181762.08FU_F_ITS2
Russula_sp_KY774272_SH1181775.08FU_F_ITS2
Russula_sp_KY774261_SH1181765.08FU_F_ITS2
Russula_sp_GQ268640_SH1181774.08FU_F_ITS2
Russula_castanopsidis_MN130078_SH1558617.08FU_F_ITS2
Russula_sp_KP071052_SH2589402.08FU_F_ITS2
Russula_castanopsidis_MN134530_SH1558629.08FU_F_ITS2
Russula_sp_MG321327_SH1558622.08FU_F_ITS2
Russula_sp_MN134536_SH3591379.08FU_F_ITS2
Russula_sp_UDB033479_SH1181757.08FU_F_ITS2
Russula_sp_KX899341_SH1181776.08FU_F_ITS2
Russula_sp_GU184104_SH2587819.08FU_F_ITS2
Russula_sp_KY774264_SH1159962.08FU_F_ITS2
Russula_lutensis_LC008293_SH1159963.08FU_F_ITS2
Russula_sp_FJ455032_SH1159961.08FU_F_ITS2
Russula_pulchra_MK564597_SH2592542.08FU_F_ITS2
Russula_sp_MK414590_SH2706847.08FU_F_ITS2
Russula_sp_LT602963_SH1538982.08FU_F_ITS2
Russula_sp_LC368013_SH1538992.08FU_F_ITS2
Russula_sp_KX619505_SH1538989.08FU_F_ITS2
Russula_sp_JF273536_SH1168703.08FU_F_ITS2
Russula_sp_MH496824_SH2592487.08FU_F_ITS2
Russula_sp_UDB057224_SH2602478.08FU_F_ITS2
0.5
