# Supplementary material for: What’s for dinner this time?: DNA authentication of “wild mushrooms” in food products sold in the USA
Source: PeerJ. 2021 Aug 2;9:e11747. doi: 10.7717/peerj.11747 (PMC8340906; doi:10.7717/peerj.11747)

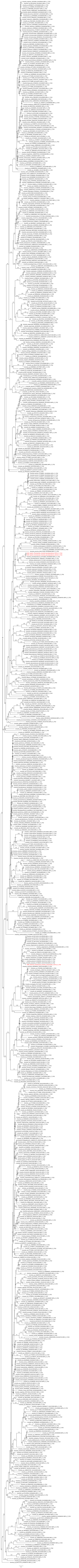

Supplement: Supplemental Information 2 [file sh_general_release_dynamic_s_10.05.2021.AMANITA.2.ITS2.mafft.fasta.treefile.2.pdf › sh_general_release_dynamic_s_10.05.2021.AMANITA.2.ITS2.mafft.iqtree/210521232900/sh_general_release_dynamic_s_10.05.2021.AMANITA.2.ITS2.mafft.fasta.treefile.2.pdf]
